# Air Bacterial Microbiomes in Hospitals: Case Studies from a Metropolis and a Small City of Thailand

**DOI:** 10.34133/csbj.0068

**Published:** 2026-04-29

**Authors:** Piraya Chatthanathon, Doonyapong Wongsawaeng, Tassanee Chetwittayachan, Thanya Cheibchalard, Asada Leelahavanichkul, Ekasit Kowitdamrong, Jarun Sayasathid, Naraporn Somboonna

**Affiliations:** ^1^ Department of Microbiology, Faculty of Science, Chulalongkorn University, Bangkok 10330, Thailand.; ^2^ Multi-Omics for Functional Products in Food, Cosmetics and Animals Research Unit, Chulalongkorn University, Bangkok 10330, Thailand.; ^3^ Department of Nuclear Engineering, Faculty of Engineering, Chulalongkorn University, Bangkok 10330, Thailand.; ^4^ Department of Environmental Science, Faculty of Science, Chulalongkorn University, Bangkok 10330, Thailand.; ^5^ Department of Microbiology, Faculty of Medicine, Chulalongkorn University, Bangkok 10330, Thailand.; ^6^ Division of Nephrology, Department of Medicine, Faculty of Medicine, Chulalongkorn University, Bangkok 10330, Thailand.; ^7^ Applied Medical Virology Research Unit, Chulalongkorn University, Bangkok 10330, Thailand.; ^8^ Cardiac Center, Naresuan University Hospital, Naresuan University, Phitsanulok 65000, Thailand.; ^9^ Omics Sciences and Bioinformatics Center, Faculty of Science, Chulalongkorn University, Bangkok 10330, Thailand.; ^10^ Microbiome Research Unit for Probiotics in Food and Cosmetics, Chulalongkorn University, Bangkok 10330, Thailand.

## Abstract

**Experimental objective:** Hospital air can act as a reservoir of opportunistic and antimicrobial-resistant microorganisms, which may contribute to hospital-acquired infections. However, the composition of airborne bacterial communities and the factors shaping them within hospital environments remain insufficiently characterized. This study investigated airborne bacterial microbiomes across hospital areas and sampling approaches and compared hospitals located in a metropolis versus a smaller city in Thailand. **Methods:** Air samples were collected from various hospital zones using active air-pump sampling and passive air-grille or high-efficiency particulate air-filter swab approaches at King Chulalongkorn Memorial Hospital in Bangkok and Naresuan University Hospital in Phitsanulok. Microbiota were analyzed using 16*S* ribosomal RNA gene sequencing, followed by bioinformatic analyses. **Results:** Bacterial community compositions and alpha-diversity varied significantly along sampling method, hospital area, and geographic location. Passive air-grille swabs captured higher microbial biomass and diversity, consistent with accumulated microbiome deposition over time. Areas with open and semiopen ventilation (e.g., restaurant and outpatient departments) exhibited higher bacterial diversity than filtered areas (e.g., operating rooms). The metropolitan hospital showed higher abundances of *Cutibacterium*, *Acinetobacter*, *Curtobacterium*, and members of Comamonadaceae, whereas the hospital in the smaller city displayed greater overall diversity. High-efficiency particulate air-filter samples showed reduced diversity but enriched in spore-forming taxa. Predicted functional profiles also differed between sampling approaches and hospital locations, including pathways that might be related with human diseases. **Conclusion:** Hospital air microbiomes were heterogeneous and influenced by environmental conditions and sampling strategy. These findings provide insights for factor correlations and may inform improved air-quality management strategies.

## Graphical Abstract

**Figure:**
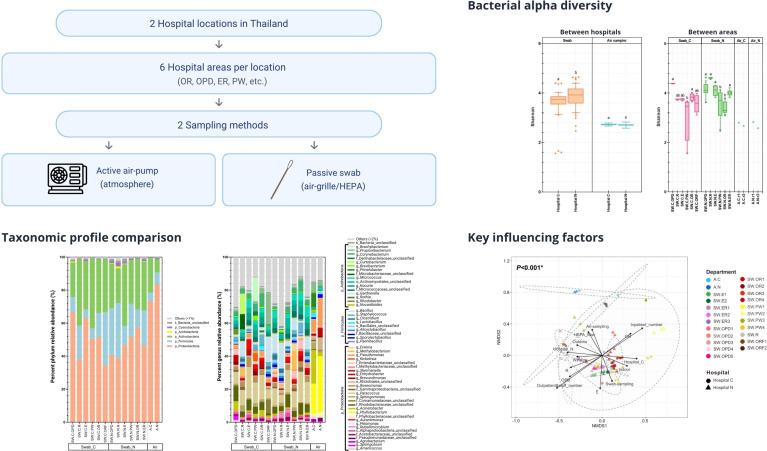

## Introduction

Air microbiome comprises predominantly bacteria (approximately 80%), then fungi, and viruses [1]. Common airborne bacteria include Pseudomonadota (i.e., families Pseudomonadales and Burkholderiales), Bacillota (i.e., Bacillales), Actinomycetota (i.e., Corynebacteriales), and Bacteroidota (i.e., Sphingobacteriales) [1,2], and general resources may be from natures and/or human–animal activities, such as soil, industries, pollutions, and agricultures [3,4]. Indoor air microbiota, particularly in hospitals, play significant impacts on human health, and hospital air may contain various opportunistic or antibiotic-resistant pathogens, such as methicillin-resistant *Staphylococcus aureus* and tetracycline-resistant *Streptococcus pyogenes* [4–6]. Studies have found airborne transmission of pathogens, antibiotic-resistant genes, and endotoxins associated with respiratory infections, meningitis, and hospital-acquired infections (HAIs) [5–9].

The bacterial diversity in buildings vary with building patterns, geographic locations, inside activities, and ventilation systems [1,10,11]. For instance, high indoor humidity correlates with increased bacteria and fungi; ventilation systems, mechanical (i.e., filter) versus natural routes, partly control air microbiota [4,11,12]. High-efficiency particulate air (HEPA) filters are generally used in hospitals to prevent pathogens; for instance, statistical differences in bacterial microbiota were reported between floor dust and HEPA filters [13–15]. Nonetheless, the indoor hospital air microbiota remain influenced by human occupancy and activities: talking, breathing, coughing, sneezing, skin–cloth–shoe shedding, any surfaces, floors, medical procedures, infectious agents, etc. [4,5,12]. Some studies reported hospital air microbiota linked to inhabited human gut, skin, saliva, and oral cavity [16].

**Fig. 1.**
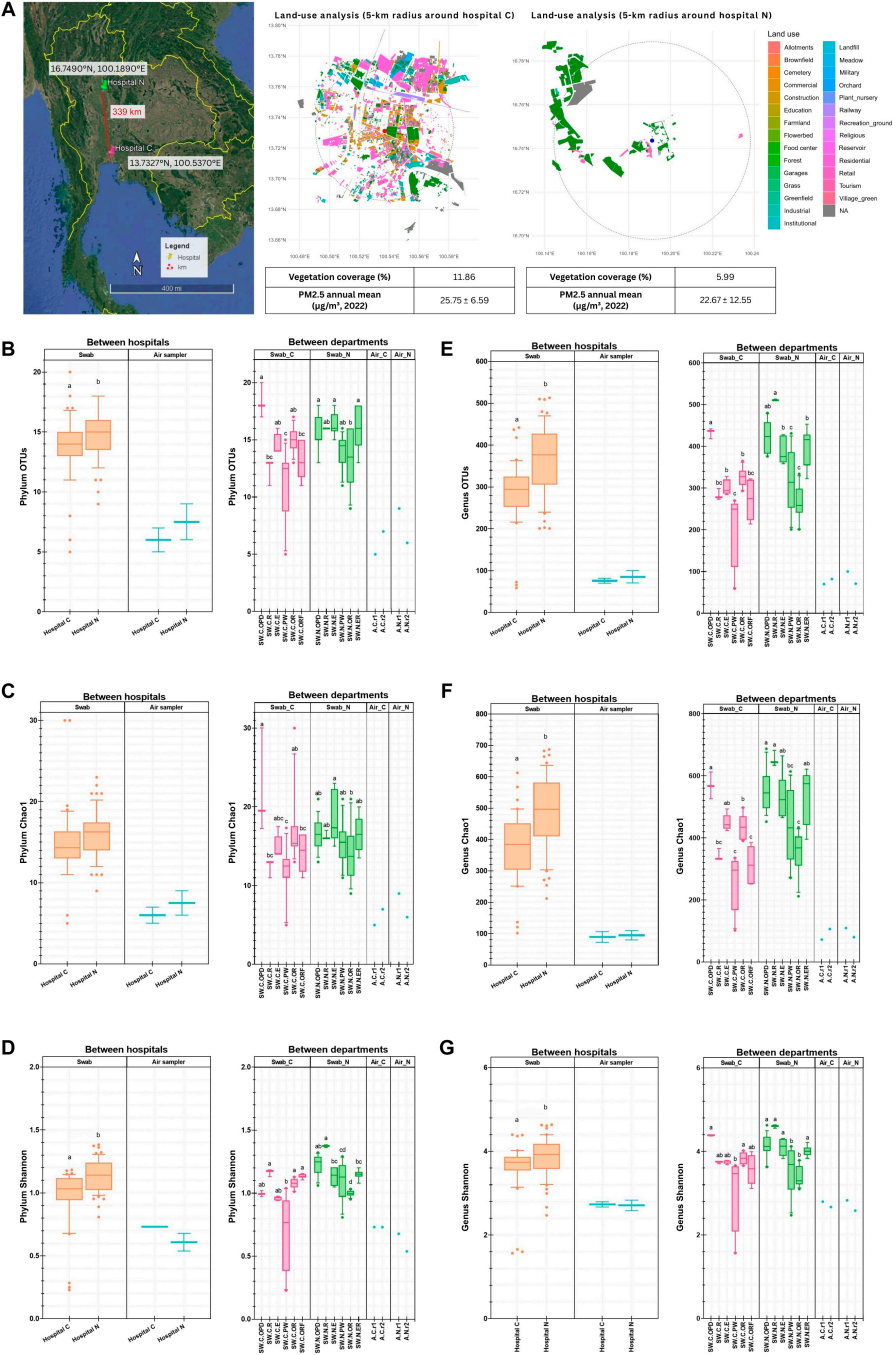
(A) Geographic map along surrounding land use, vegetation coverage and PM2.5 (particulate matter ≤ 2.5 μm in diameter) levels, and alpha-diversity indices at phylum [(B) operational taxonomic units (OTUs), (C) Chao1, and (D) Shannon] and genus [(E) OTUs, (F) Chao1, and (G) Shannon] levels, comparing between hospitals C and N in aspects of passive filter-swab samples, different areas, and active air-pump samples. Statistical comparisons in regard to the average data between hospitals were performed via unpaired 2-tailed *t* test, and different alphabet letters represent statistically significant, *P* < 0.05. Similarly, for comparisons among different areas, ANOVA with Tukey’s honestly significantly different (HSD) was used instead.

This study examines hospitals in Thailand’s tropical monsoon regions, comparing urban and rural locations (Fig. 1A; differences in surrounding land use [11.86%], vegetation coverage [5.99%], and PM2.5 (particulate matter ≤ 2.5 μm in diameter) were 25.75 ± 6.59 and 22.67 ± 12.55 μg/m^3^ for King Chulalongkorn Memorial Hospital [hospital C] and Naresuan University Hospital [hospital N], respectively), different hospital areas, and passive air-grille swab versus active air-pump sampling methods. In metropolis such as Bangkok, pollution including PM2.5 could act as carriers and habitats for microbes and, hence, may influence the increased airborne abundances and diversity [1,11,17,18]. Pathogens and antibiotic resistance genes were reported to be more prevalent in PM-polluted air; yet, severe climate pollutions sometimes showed reduced microbial diversity, as the pollutions might pose toxic and cellular damage to bacteria [19,20]. Vegetation loss in Bangkok also affected airborne bacterial diversity, and researches highlighted that green spaces may lower bacterial pathogens [17,21–23]. Subsequently, airborne bacterial microbiomes in Thai hospitals located in metropolis versus small city may be different.

This study collected air samples via active air-pump (flow rate of 100 l/min based on previous literature [24,25]) and passive air-grille swab sampling from ventilation systems, from different areas in individual buildings of a metropolis hospital C and a small city hospital N, and performed 16*S* ribosomal RNA (rRNA) gene sequencing to bioinformatically analyze bacterial communities. The passive air-grille swab sampling method yielded higher microbial density (metagenomic DNA per nanogram) and diversity than the active air-pump sampling method. The relatively low air microbial density in indoor hospital air, observed through active air-pump, could relate to the standard clean-air management practice for ventilation in hospitals; consequently, passive air-grille swabs may serve as an appropriate sampling technique to capture hidden bacterial diversity in indoors [26–28]. Further, we compared microbiota’s correlations with environmental factors in individual geographies, hospital areas, and human indoor activities and associated the findings with estimated microbial metabolic functions. Collectively, this study advances our understanding of hospital air microbiomes in Thailand and contributes to increased awareness of health-related issues surrounding airborne microbial exposure, infection control, and hospital air quality. The findings also confirm safety and suggest venues for hospital management to maximize air quality for patients and clinical personnels.

## Results

### Metagenomic DNA concentration per unit area across samples

The metagenomic DNA concentrations of airborne microorganisms collected by active air-pump sampling (100 l/min) were averagely 1.10 ± 0.11 ng/m^3^ in hospital C and 7.01 ± 0.98 ng/m^3^ in hospital N, and passive air-grille swab sampling yielded on average 346.016 ± 207.194 ng/m^2^ in hospital C and 359.597 ± 271.987 ng/m^2^ in hospital N, respectively (Table S1) (note that the sample-naming convention is explained in Table S1). The differences of metagenomic DNA between sampling methods were statistically significance (*P* < 0.05). As active air-pump samples in hospitals contained too small metagenomic DNA concentrations to continue for the 16*S* rRNA gene library preparation, the independent replicate samples of various areas in the same hospital were pooled to obtain sufficient concentration for sequencing (Table S1; e.g., A.C.r2 and A.N.r2). For passive air-grille swab samples, the metagenomic DNA concentrations of individual replicates were sufficient for sequencing and were likely low in area examination rooms (ERs).

### 16*S* rRNA gene sequencing results

A total of 102 samples were sequenced, with an average of 56,664 quality reads per sample (Table S1): The number of quality reads ranged from 22,209 to 47,589 for active air-pump samples in hospital C (abbreviated A.C.r1 and A.C.r2); 17,913 to 36,673 for active air-pump samples in hospital N (A.N.r1 and A.N.r2); 15,448 to 59,990 for passive air-grille swab samples in hospital C (abbreviated SW.C); and 7,703 to 100,221 for passive air-grille swab samples in hospital N (abbreviated SW.N). Note that for swab samples, letters following SW.N and SW.C represent different hospital areas (outpatient department [OPD], restaurant [R], elevator [E], patient wards [PWs], operating room [OR], ER, and HEPA filter in operating room [ORF]), and r1, r2, or r3 represent independently replicate sample 1, 2, or 3, respectively. All samples were normalized to equal sequencing depth (12,041 quality reads per sample), where Good’s coverage indices ranged from 99.95% to 100% at phylum and 98.57% to 99.96% at genus levels (Table S2). Rarefaction curves are reaching plateau at phylum level (Fig. S1).

### Alpha-diversity indices differed between sampling methods, between metropolis and small city locations, and among hospital areas

Alpha-diversity of bacterial communities was analyzed via observed operational taxonomic units (number of OTUs), Chao1 (OTU richness), and Shannon (OTU evenness). Passive air-grille swab samples in hospital N exhibited generally statistically higher alpha-diversity than those in hospital C (Fig. 1B and D [e.g., phylum level: *P* = 0.01 for OTUs and *P* < 0.0001 for Shannon] and Fig. 1E to G [genus level: *P* < 0.0001 for OTUs, *P* < 0.0001 for Chao1, and *P* = 0.02 for Shannon]), and active air-pump samples yielded clearly lower alpha-diversity than passive air-grille swab samples (Fig. 1B to G and Table S2). In aspects of areas in a hospital building, areas near entrance (OPD and R) exhibited generally higher alpha-diversity than enclosed indoor areas (ER, E, OR, and PW).

**Fig. 2.**
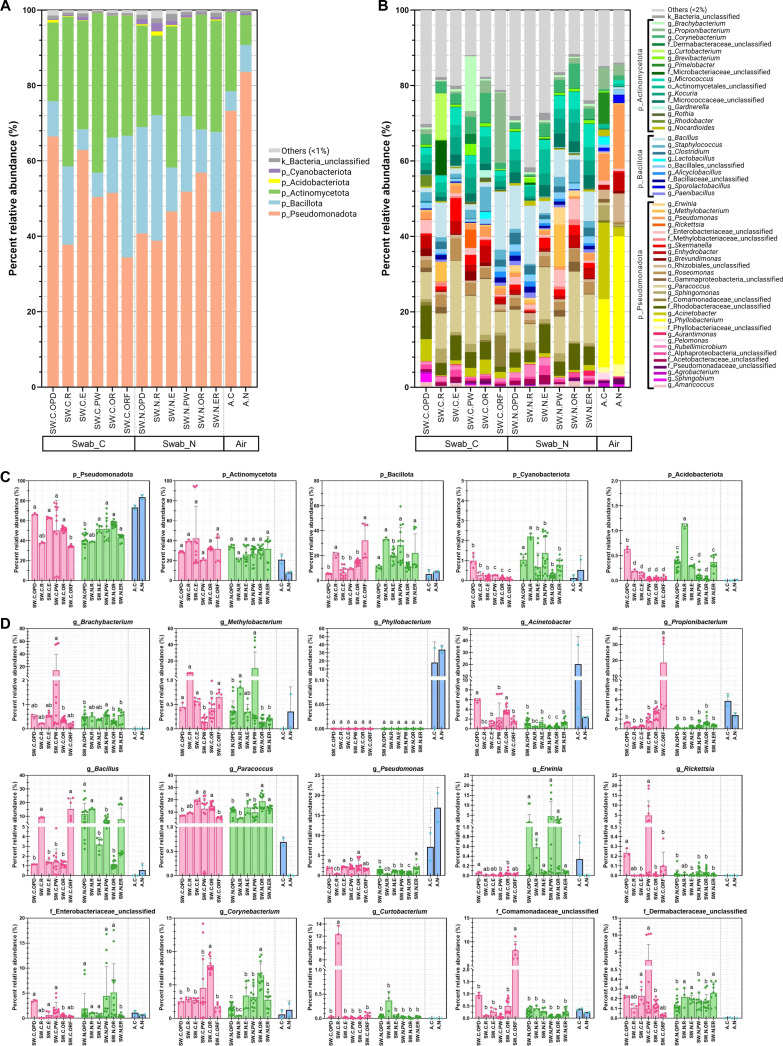
Average percent relative compositions in (A and C) phylum and (B and D) genus levels, with (C) displayed the top 5 phylum statistics and (D) displayed the top 15 genus statistics. k_, kingdom; p_, phylum; c_, class; o_, order; f_, family; g_, genus; others, phyla (or genera) with <1% (or 2%). Statistical comparisons were performed via ANOVA with Tukey’s honestly significantly different (HSD), and different alphabet letters represent statistically different (*P* < 0.05).

Details into the profiles of bacterial compositions revealed a total of 37 bacterial phyla, with 5 predominant phyla including Pseudomonadota, Actinomycetota, Bacillota, Cyanobacteriota, and Acidobacteriota (formerly Proteobacteria, Actinobacteria, Firmicutes, Cyanobacteria, and Acidobacteria, respectively) comprising >99% (Fig. 2A and Fig. S2A). Among active air-pump samples, phyla Pseudomonadota and Bacillota were lower, while Actinobacteria were higher in hospital C than in hospital N (*P* = 0.061 for Pseudomonadota, *P* = 0.588 for Bacillota, and *P* = 0.186 for Actinomycetota). For passive air-grille swab samples, comparative differences between hospitals in different areas were also analyzed. Analyses at genus composition revealed 1,277 OTUs in total, and 54 predominant genera were from Pseudomonadota, Actinomycetota, and Bacillota (Fig. 2B and Fig. S2B). For example, Pseudomonadota were relatively greater in hospital C areas OPD and E, whereas Bacillota were lower in hospital C areas OPD and PW (Fig. 2C).

Analysis of the top 15 genera across areas and hospitals showed many significant differences: For instance, *Acinetobacter* and *Paracoccus* were greater in A.C. than in A.N., but the differences were not statistically significant, while Pseudomonadota, *Cutibacterium* (*Propionibacterium*), and *Pseudomonas* were lower in A.C. than in A.N., although not significant. In passive air-grille swab samples, while some bacterial genera seemed relatively consistent across areas, certain genera such as *Methylobacterium*, *Brachybacterium*, *Paracoccus*, and *Corynebacteriu*m were relatively different across areas (*P* < 0.05) (Fig. 2C and D). Notably, *Bacillu*s and *Erwinia* were relatively high in hospital N across areas; meanwhile, *Bacillus* were high in hospital C areas R and ORF. In addition, *Propionibacterium* was significantly higher in hospital C area ORF, *Rickettsia* was higher in C areas OPD and PW, and *Curtobacterium* was higher in C area R (although within hospital N, *Curtobacterium* was highest in R but to a much smaller degree) (Fig. 2D).

### Beta-diversity analyses and effects by locations and hospital areas

**Fig. 3.**
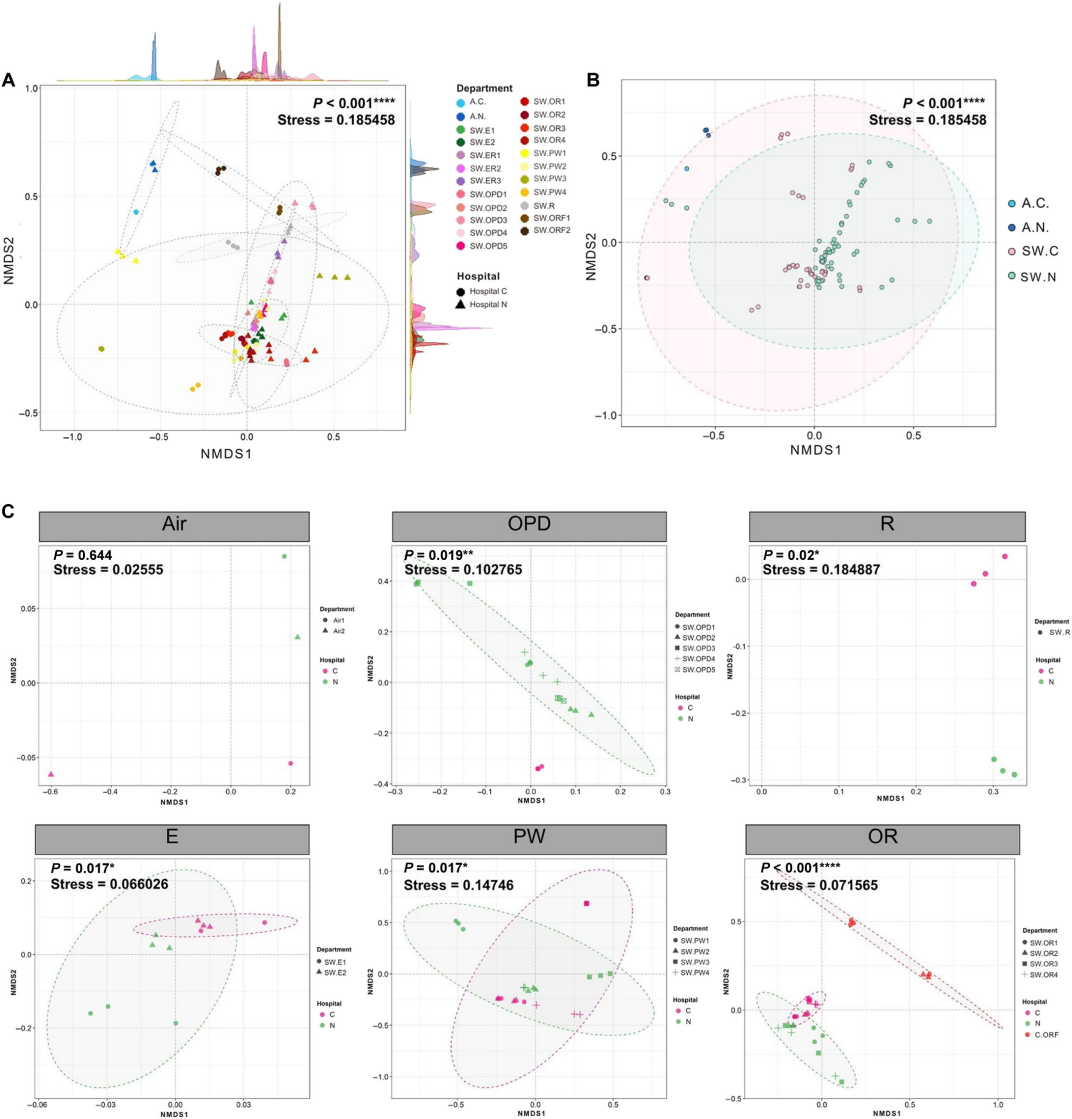
Nonmetric multidimensional scaling (NMDS) constructed by Morisita–Horn dissimilarity indices showing relationships among genus level microbiota along 90% confidence clustering via multivariate normal distribution: (A) comparisons among hospital areas and sampling methods, (B) average between hospitals C and N, and (C) between hospitals C and N by single variations (air pump sampling and areas in hospitals). In (A), density plots along axes illustrated similarities in relationships. Statistical comparisons were performed via AMOVA with asterisks denoting significance levels (*P < 0.05, **P < 0.01, ***P < 0.001, and ****P < 0.001).

Beta-diversity analyses of all samples showed that the microbiota were statistically distinct by area (*P* < 0.001) and the diversity among active air-pump samples (A.C. and A.N.) was minor and clustered separately from passive air-grille swab samples (SW), reflecting data from atmospheric air versus ventilation system microbiota (Fig. 3A and B). Figure 3B indicated *P* < 0.001 for statistical differences of active and passive air sample microbiota between C and N, and detailed pairwise statistics indicated that actual difference was between SW.N and SW.C (Table 1). Among passive air-grille swab samples, area PW microbiota were rather diverse among samples (Fig. 3A). Air-grille swab microbiota varied significantly across most hospital areas, except between ER and OPD and between ER and PW (Table 1). However, hospital locations (C or N) played role in diversity: Microbiota of OPD, R, E, PW, and OR clustered separately by location (C or N) (Fig. 3C; *P* = 0.019, *P* = 0.020, *P* = 0.017, *P* = 0.017, and *P* < 0.001, respectively). Note that the SW.C.ORF (ORF represents HEPA filter-swab microbiota in hospital C area OR) seemed close to SW.N.OR microbiota, but the analysis of molecular variance (AMOVA) statistic remained significantly different (Table S3; *P* < 0.001).

**Table 1.** AMOVA statistics of beta-diversity Morisita–Horn dissimilarity indices comparing hospital areas, sampling methods, and between hospitals C and N. *P* < 0.05 indicates statistical difference, and ORF was excluded in pairwise area comparisons because these samples were limited to hospital C.

Pairwise hospital areas	*P* value
E versus ER	0.009
E versus OPD	0.002
E versus OR	<0.001
E versus PW	0.018
E versus R	<0.001
ER versus OPD	0.322
ER versus OR	<0.001
ER versus PW	0.08
ER versus R	0.001
OPD versus OR	<0.001
OPD versus PW	0.001
OPD versus R	0.016
OR versus PW	<0.001
OR versus R	<0.001
PW versus R	0.01
**Pairwise sampling methods**	***P* value**
Air pump versus filter swab	<0.001
**Pairwise hospital locations**	***P* value**
A.C. versus A.N.	0.644
SW.N versus SW.C	<0.001

ORF, high-efficiency particulate air filter in operating room; E, elevator; ER, emergency room; OPD, outpatient department; OR, operating room; PW, patient ward; R, restaurant.

### Analyses of potential influencing factors, representing bacterial taxa, and LEfSe species biomarkers

**Fig. 4.**
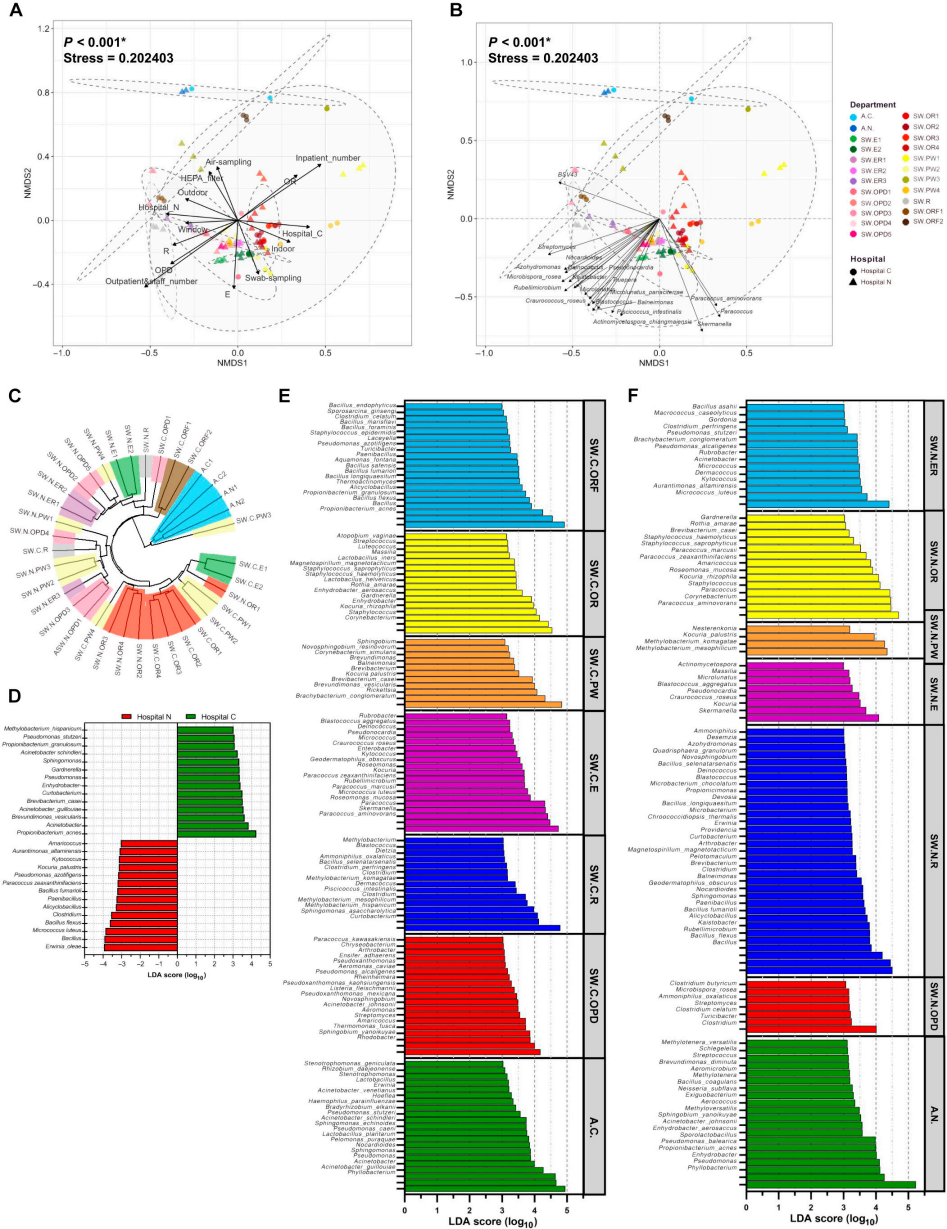
Nonmetric multidimensional scaling (NMDS) (by Morisita–Horn dissimilarity indices) and Spearman’s correlations with (A) microbiota-influencing factors (*P* < 0.05); (B) predominant bacterial taxa (*P* < 0.05); (C) alternative visualization of sample clustering via phylogenetic tree (by Jaccard indices) denoting same hospital areas with same colors; and LEfSe (linear discriminant analysis effect size) for species biomarkers (*P* ≤ 0.05 and LDA score > 3.0) when comparing (D) between hospitals C and N, (E) across areas in hospital C, and (F) across areas in hospital N. All data were analyzed at species operational taxonomic unit (OTU) levels. In (A) and (B), ellipses represent 90% confidence clusters from multivariate normal distribution, arrows indicate direction and magnitude of correlations, and statistical differences were computed using AMOVA.

Spearman’s correlations were analyzed to determine potential microbiota-influencing hospital factors, including sampling methods, hospital locations (associating microbiota in opposite directions), inpatient numbers, outpatient and staff numbers, distance and number of doors and windows (for some area types), area types (except areas PW [*P* = 0.093] and ER [*P* = 0.165]), and presence of HEPA filter (Fig. 4A; *P* < 0.05). Opposite arrow directions were observed between air and swab sampling, hospitals C and N, or in- and out-patient (including staff) numbers. Microbiota-influencing area types differ, ranging from relative distances with outdoor area, windows to crowded outpatient areas, limited access areas, and operating areas with HEPA filters. HEPA filter factor showed correlation in a direction of ORF and active air-pump microbiota. Many predominant bacteria species were represented and along the relatedness with outpatient numbers and crowded area types (areas E, ER, OPD, and R) (*P* < 0.05) (Fig. 4A and B).

Phylogenetic clustering confirmed the similarity of microbiota structures based on the sampling methods, relative hospital location, and area types, respectively. Clear clustering was observed within active air-pump microbiota, OR microbiota, and ORF microbiota (Fig. 4C). However, the phylogenetic analysis showed that microbiome structures were altered along area types, followed by hospital location, consistent with Fig. 4A.

LEfSe (linear discriminant analysis effect size) identified species markers differentiating hospital locations (species reports from the 16*S* rRNA gene hypervariable regions V3 to V5 represented best-match assignments; meanwhile, the genus-level resolution was more reliable) [29] (Fig. 4D; *P* < 0.05): for hospital C such as *Propionibacterium* (new genus name *Cutibacterium*) *acnes* (LDA score = 4.26), *Brevundimonas vesicularis* (3.63), *Acinetobacter guillouiae* (3.56), *Brevibacterium casei* (3.56), *Acinetobacter schindleri* (3.25), *Propionibacterium granulosum* (3.10), *Pseudomonas stutzeri* (3.08), and *Methylobacterium hispanicum* (3.03) (average LDA score = 3.45) and for hospital N such as *Erwinia oleae* (LDA score = 3.95), *Micrococcus luteus* (3.87), *Bacillus flexus* (3.63), *Bacillus fumarioli* (3.23), *Paracoccus zeaxanthinifaciens* (3.22), *Pseudomonas azotifigens* (3.19), *Kocuria palustris* (3.16), and *Aurantimonas altamirensis* (3.11) (average LDA score = 3.40). These results were consistent with Fig. 2B and D. In the same hospital, we also analyzed species signatures for each area type (Fig. 4E and F): Shared species for certain areas, albeit in different hospitals (C or N), were found and reflected similar activities of those areas (for example, *Kocuria rhizophila*, *Rothia amarae*, *Staphylococcus haemolyticus*, and *Staphylococcus saprophyticus* in OR; *Blastococcus* in E; and *Streptomyces* in OPD). Moreover, many species marker in SW.C.ORF are spore-forming bacteria (e.g., *B. flexus*, *Thermoactinomyces*, *Bacillus longiquaesitum*, *Paenibacillus*, *Clostridium celatum*, *Sporosarcina ginsengi*, and *Lysinibacillus*) (average LDA score = 2.88 for hospital C and 2.78 for hospital N) (Fig. 4E).

### Potential microbial metabolic profile analyses

**Fig. 5.**
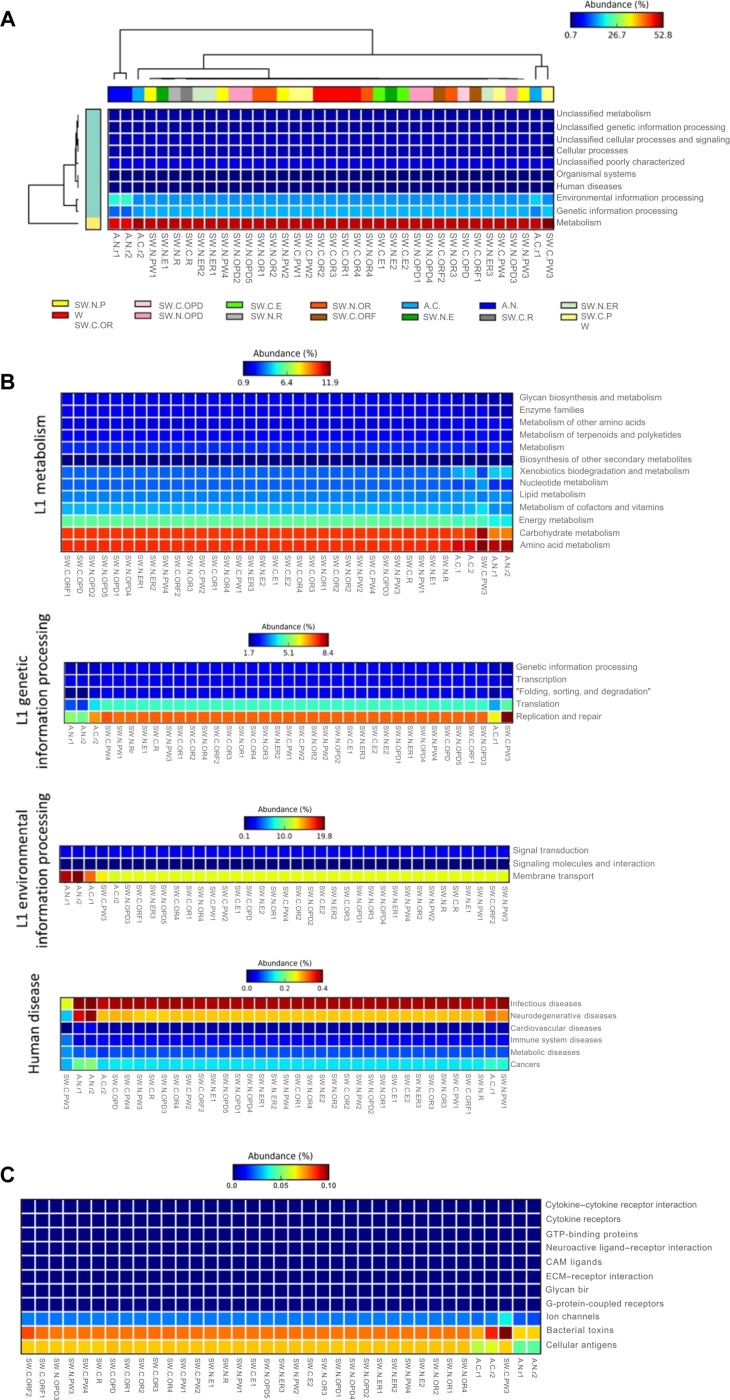
Heatmaps representing estimated microbial metabolic profiles based on Kyoto Encyclopedia of Genes and Genomes (KEGG) pathways in (A) level 1, (B) level 2 (subcategories into metabolism pathways, genetic information processing pathways, environmental information processing pathways, and human disease pathways), and (C) level 3 (signaling molecules and interaction channels). Dendrograms were computed using unweighted pair group method with arithmetic mean (UPGMA) with default parameters and threshold 0.75. Percent relative abundance of each metabolic is in gradient color from blue (low) to red (high abundances). In (B), each functional inference contained in a parenthesis the nearest sequenced taxon index (NSTI) values to justify PICRUSt (Phylogenetic Investigation of Communities by Reconstruction of Unobserved States) reliability, provided that the lower NSTI (e.g., <0.15) inferred higher accuracy.

The potential microbial-related metabolic profiles following Kyoto Encyclopedia of Genes and Genomes (KEGG) pathway level 1 clustered active air-pump microbial metabolic profiles of A.N., followed by A.C. (and SW.C.PW3), separately from others that represented passive air-grille swab profiles of different hospitals (locations) and areas. The A.N. exhibited the greater environmental information processing than A.C. (and also passive swab samples) but reduced genetic information processing and metabolism compared with A.C. (Fig. 5A). Note that the active air-pump results were descriptive (no statistic comparison could be performed limited to only 2 replicates). At KEGG level 2, subcategories of pathways in level 1, various metabolisms in air-pump samples (i.e., carbohydrate metabolism; replication and repair; translation; folding, sorting, and degradation; transcription; membrane transport; energy metabolism; metabolism of cofactors and vitamins; nucleotide metabolism; enzyme families; and glycan biosynthesis and metabolism) were reduced, while minor particular metabolisms that increased included xenobiotics biodegradation and metabolism and amino acid metabolism, and, interestingly, the differences were also observed in the human-disease-related subcategory of metabolisms (Fig. 5B). The more prominent subcategories of related human disease in air microbial profiles were annotated under pathways related to neurodegenerative diseases, cardiovascular diseases, and cancers, and A.N. showed higher levels of pathways related to infectious diseases, neurodegenerative diseases, cardiovascular diseases, metabolic diseases, and cancers compared to A.C. On the other hand, passive air-grille swab profiles were annotated for relatively higher in immune system diseases. In KEGG level 2, each functional inference was included the nearest sequenced taxon index (NSTI) value to justify its PICRUSt (Phylogenetic Investigation of Communities by Reconstruction of Unobserved States) reliability. Further analyses of signaling molecules and interaction channels (Fig. 5C) showed that few statistical differences were found but cellular antigens and bacterial toxins were found relatively higher in passive air-grille swab profiles.

### Microbiomes in the OR were associated with specific bacteria, surgery activities, and HEPA filter

**Fig. 6.**
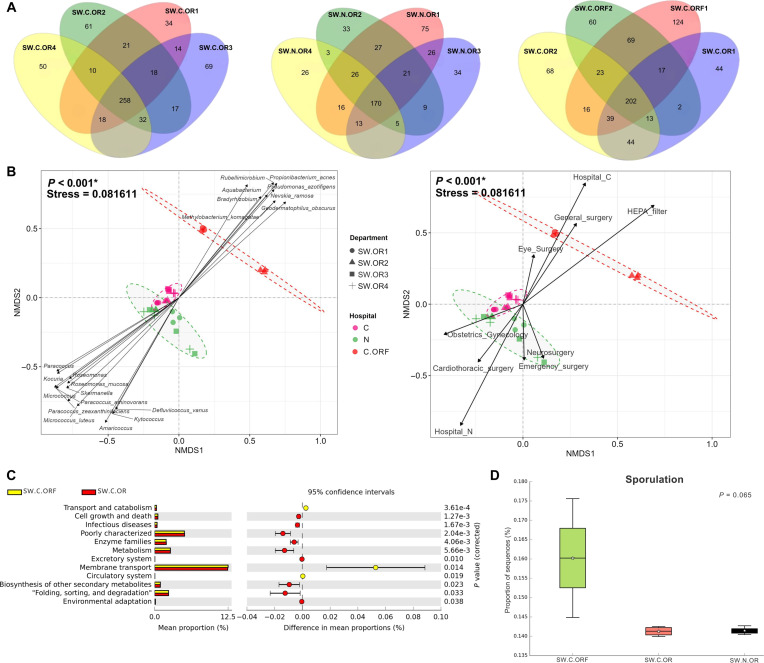
Microbiome alterations in the operating room are influenced by specific bacteria, surgery activities and the high-efficiency particulate air (HEPA) filter: (A) Venn diagrams of the operating room (OR) microbiomes at species level operational taxonomic units (OTUs) (hospital C, hospital N, and SW.C.OR versus SW.C.ORF in hospital C); (B) nonmetric multidimensional scaling (NMDS) (by Morisita–Horn dissimilarity indices) and Spearman’s correlations to identify representative species and OR activities (AMOVA, *P* < 0.05); (C) differential estimated microbial metabolisms Kyoto Encyclopedia of Genes and Genomes (KEGG) level 2 between SW.C.OR and SW.C.ORF (2-sided *t* test, *P* < 0.05); and (D) KEGG level 3 (box plot of percent relative sporulation) (ANOVA). In (B), only top 20 OTUs classified as genus and species were displayed, ellipses represent 90% confidence clusters from multivariate normal distribution, and arrows indicate direction and magnitude of statistical correlation factors.

Venn diagrams exhibited the number of shared and unique bacterial species in OR of hospital C (258 shared OTUs and on average 53.5 unique OTUs per sample) and hospital N (170 shared OTUs and on average 42 unique OTUs per sample) (Fig. 6A). In detail, 34, 61, 69, and 50 OTUs were unique to OR1 (mainly general surgery), OR2 (general surgery), OR3 (eye surgery), and OR4 (eye surgery) in hospital C; and 75, 33, 34, and 26 OTUs were unique to OR1 (mainly neurosurgery), OR2 (obstetrics and gynecology), OR3 (emergency surgery), and OR4 (cardiothoracic surgery) in hospital N, respectively. Figure 6B displayed associated species with areas OR of hospital C versus hospital N, and the unique representative species in SW.C.ORF samples along the same direction as SW.C.OR, with no statistical difference between SW.C.OR1 and SW.C.ORF1 (Table S3B; *P* = 0.124) and between SW.C.OR2 and SW.C.ORF2 (*P* = 0.098). However, following the results showed that HEPA filters contained selectively bacterial species (Fig. 4E). The top 20 classified genera that represented these microbiota structures were plotted, revealing that *Geodermatophilus obscurus*, *Propionibacterium acnes*, *Methylobacterium komagatae*, *P. azotifigens*, *Rubellimicrobium*, *Nevskia ramosa*, *Bradyrhizobium*, and *Aquabacterium* were more positively correlated with SW.C.ORF and *Kytococcus*, *Amaricoccus*, *M. luteus*, *Roseomonas*, *Micrococcus*, *Roseomonas mucosa*, *Skermanella*, *Kocuria*, *Paracoccus*, *P. zeaxanthinifaciens*, *Paracoccus aminovorans*, and *Defluviicoccus vanus* were more correlated with SW.C.OR and SW.N.OR microbiota (Fig. 6B). We also identified the significant correlations with the OR activities. Meanwhile, hospital locations (C or N) (*P* < 0.001, vector length = 0.91) and HEPA filter (*P* < 0.001, length = 0.98) showed the strong impact, followed by the OR activities (*P* < 0.05 and in relative order of vector lengths: general surgery, cardiothoracic surgery, obstetrics and gynecology, neurosurgery, emergency surgery, and eye surgery) (Fig. 6B).

The estimated microbial metabolic profiles of SW.C.OR and SW.C.ORF were compared and revealed that the HEPA microbiota had reduced functions related to metabolism; folding, sorting, and degradation; biosynthesis of other secondary metabolites; cell growth and death; and infectious disease (Fig. 6C). For the sporulation pathway, the SW.C.ORF significantly increased compared with SW.C.OR and SW.N.OR (*P* = 0.065), consistent with the higher abundance of spore-forming bacteria in the HEPA filter (Fig. 4E).

## Discussion

Hospital indoor air represents a critical interface between the built environment, human occupants, and microbial exposure. Compared with most indoor settings, hospitals are characterized by high human traffic, prolonged occupancy, frequent medical procedures, and intensive use of antibiotics and disinfectants. These factors collectively increase the likelihood that hospital air harbors opportunistic or antimicrobial-resistant microorganisms, thereby contributing to HAIs [4–6,30]. The present study provided a comprehensive comparison of airborne bacterial microbiomes across hospital areas, sampling strategies, and geographic locations in Thailand and offers new insights into how abiotic and biotic factors affect the hospital indoor air microbiomes. This knowledge helps to improve indoor air quality and respiratory safety for patients, staff, and visitors.

The 16*S* rRNA gene sequencing was successful as the number of quality reads qualified the high Good’s coverage indices. Rarefaction to equal sequencing depth per sample was selected to standardize sequencing power across samples with variable read depth, consistent with ecological diversity comparisons [31]. Although rarefaction is increasingly discouraged because of potential data loss, it remains appropriate for standardizing sequencing effort in comparative analyses of community diversity where the selected rarefaction depth retained high Good’s coverage (>98.5% at genus level), indicating adequate representation of microbial diversity. As our focus was on alpha- and beta-diversity patterns rather than differential abundance testing, rarefaction provided a pragmatic normalization approach. Other alternative methods (e.g., variance-stabilizing approach) may further improve sensitivity in future studies.

Clear differences in bacterial community composition and alpha-diversity were observed between hospitals located in a metropolis (Bangkok) and a smaller city (Phitsanulok), particularly in passive air-grille swab samples. The active air-pump samples showed weaker geographic separation, perhaps reflecting lower biomass and the pooling of replicates required to obtain sufficient DNA for sequencing, which limited the statistical comparisons across groups as well as the parallel analyses between the sampling approaches. Consistent with previous studies (i.e., various microbiota among urban, suburban, and rural homes [32] and dust swab sampling microbiota showed diverse biodiversity even among different hotel rooms [33]), here, we hypothesized that urbanization level, which included surrounding vegetation and air pollution (i.e., annual mean local PM2.5 of 25.75 ± 6.59 μg/m^3^ for hospital C and 22.67 ± 12.55 μg/m^3^ for hospital N during the study year 2022 [34], municipal vegetation coverage of 11.86% for hospital C and 5.99% for hospital N, and land-use classifications or representative site photographs) (Fig. 1A, Fig. S3, and Table S4), affected indoor microbiomes. Dust- or surface-associated samples allow microbial inputs over longer time scales than active air-pump samples, which might explain their greater capture of metagenomic concentrations and hence the clearer geographic effects, as well as the air ventilation systems as a source of airborne pathogens and toxins that could impact human health. The lower bacterial alpha-diversity observed in hospital C was in line with ecological studies that urbanization tended to reduce environmental microbial diversity [33,35,36]. Hospital C exhibited significantly higher urbanization and anthropogenic pressure than hospital N, including population density of 3,501 versus 84 persons/km^2^ [37–40], commercial (red zone) versus agricultural (green zone) land use (Fig. S3) [41,42], and approximately 5-fold higher outpatient visits (annually 1.84 million versus 362,480), 3-fold higher inpatient capacity (1,427 versus 485 beds), and admissions (45,539 versus 14,531 patients) (Table S4) [43,44]. In contrast, hospital N in smaller city or less urbanization might receive more diverse microbial inputs from surrounding natural environments through natural ventilation (e.g., windows and doors) and human movements. The distinct clustering of samples by hospital location within comparable areas further emphasized that regional environmental context acts as an important background shaping hospital microbiome. As a limitation, the study lacked field or metagenomic extraction blank (extracting metagenome from the sterile filer or swab) as a negative control.

Among all variables examined, hospital areas emerged as the strongest determinant of bacterial community structures. Departmental areas differ in occupant density, patient turnover, activity type, access control, and ventilation strategy, all of which influence microbial dispersal and persistence [4,5,12,45]. This finding aligns with earlier studies. For example, research conducted in Brazilian hospitals showed that the microbiomes collected through swab sampling varied across different areas within each hospital. However, the study found no statistically significant differences in microbial diversity between the hospitals themselves, likely because all the hospitals were situated within the same geographic region [46]. In addition, we found higher alpha-diversity in naturally ventilated or semiopen areas, such as OPD and R, compared to mechanically ventilated areas (e.g., E, PW, OR, ER, and ORF). Window and door ventilation showed a significant association with microbiome composition (AMOVA, *P* < 0.05), supporting the role of outdoor air exchange in enhancing microbial diversity [47]. Mechanical ventilation, while effective at reducing airborne particle loads, also creates more homogeneous and lower diversity microbial communities, e.g., by filtration. Nonetheless, improperly maintained ventilation system might act as a source of contamination and microbial concentration [14,47–49]. The findings highlighted that for within the building, the ventilation type and maintenance, and hospital area strongly influence microbial diversity and composition. Similarities observed among ER, OPD, and PW swab samples likely reflected spatial proximity, shared airflow patterns, and overlapping human traffic, highlighting how hospital layout and functional connectivity might facilitate microbiome convergence and potentially cross-area microbial exchange [50].

Following key bacterial taxa and potential clinical relevance, Pseudomonadota dominated the bacterial communities, followed by Actinomycetota, Bacillota, and Cyanobacteriota, consistent with previous hospital and indoor air microbiome studies [46,51]. Biomarker analyses revealed taxa associated with specific sampling methods, hospital locations, and areas. Air-pump samples were enriched in phyllosphere-associated bacteria such as *Phyllobacterium* and *Pseudomonas*, reflecting inputs from surrounding vegetation and outdoor air, and these bacteria are generally resistant to extreme environments (i.e., low moisture, low nutrient availability, high temperature, and ultraviolet radiation) [52,53]. Although many phyllosphere bacteria are environmentally benign, certain species have been implicated in opportunistic infections, underscoring the need to consider ecological origin alongside clinical relevance [54,55].

ORs formed a rather distinct microbiome cluster across both hospitals. *Staphylococcus* genus (such as *S. haemolyticus* and *S. saprophyticus*) was identified as biomarker, consistent with reports that staphylococci are well adapted to cleanroom environments and can persist despite routine disinfection agents or some antibiotics (i.e., vancomycin) [56]. In addition, some species of *Staphylococcus* can produce biofilm, supporting their resistance or tolerance to disinfectants or antibiotics [56]. Nevertheless, although particular species in this genus could potentially be pathogenic (e.g., *Staphylococcus*, *S. haemolyticus*, and *S. saprophyticus*), overall airborne microbial biomass in ORs was generally very low because of air-handling practices and controls [57,58], highlighting that the continued surveillance and maintenance of ventilation systems, cleaning, and access controls are important.

HEPA filtration exerted as a strong ecological filter and thus influence on microbial community structure [58,59]. HEPA filters remove 99.97% of particles of >0.3 μm. HEPA-filtered ORs likely displayed reduced diversity [60]. The high similarity between microbiomes from different OR rooms (with surgical type variations and number of staff) and hospitals suggested that filtration systems partly homogenized microbial communities, consistent with previous reports [58,59]. Further, we found that the ORF (HEPA filter) samples were enriched in spore-forming bacteria such as *Bacillus*, *Paenibacillus*, *Clostridium*, and *Lysinibacillus*. These taxa are well known for resistance to desiccation, nutrient limitation, and chemical stress, enabling long-term persistence within filter materials. While HEPA filters effectively reduce airborne microbial loads, trapped microorganisms are not necessarily inactivated. Accumulated dust and organic matter on filters might provide microhabitats that support microbial survival and, potentially, growth [14,33]. Reaerosolization during filter maintenance therefore represents a plausible exposure route [61], emphasizing the importance of regular filter replacement, appropriate handling, and complementary disinfection strategies.

Predicted functional analyses revealed clear differences between active air-pump (a snapshot of air microbiome at collection time) and passive air-grille swab (accumulated air) microbiomes. Air-pump samples exhibited reduced representation of metabolic and genetic information processing pathways, consistent with physiological stress experienced by microorganisms in the airborne state [62]. In contrast, swab samples showed higher relative abundances of annotated pathways related to cellular antigens and bacterial toxins, suggesting that the surface-associated air systems might act as reservoirs for biologically active microbial components [62,63]. Notably, the smaller-city hospital N showed higher inferred abundances of pathways related to infectious and other human diseases compared with the metropolitan hospital C. However, such functional predictions were annotated from the microbiota data, which remained indirect and did not equal functional gene expression study. The KEGG “Human disease” categories reflected pathway annotations, not pathogenicity. This functional inference required experimental validation.

Regarding the statistical robustness, we conducted both parametric (Student’s *t* test and analysis of variance [ANOVA]) (Figs. 1B to G and 2C and D) and nonparametric equivalents (Mann–Whitney *U* and Kruskal–Wallis tests with false discovery rate [FDR] correction) (Figs. S4 and S5). Alpha-diversity comparisons between hospitals yielded concordant results across both statistical approaches. Passive air-grille swab samples exhibited significantly higher OTU richness, Chao1 index, and Shannon diversity in hospital N compared to hospital C at phylum and genus levels (*P* < 0.05; Fig. 1B and Fig. S4). Similarly, among departmental areas, both ANOVA and Kruskal–Wallis tests identified identical significant differences in alpha-diversity (Fig. 1B and Fig. S4) and bacterial abundance across departments (*P*_adj_ < 0.05) (Fig. 2C and D and Fig. S5). For instance, p_Bacillota showed significantly higher relative abundance in R and ORF of hospital C and in R, PW, and E of hospital N across both statistical computations (Fig. 2C and D and Fig. S5). The consistency of statistical significance across test types strengthens confidence in these biological differences and supports no violation of data normalization as a confounding factor.

To address consistency of the bioinformatic pipeline results, we compared our analyses by OTU-based clustering (SILVA 138.1 alignment, Greengenes 13.8.99 classification) with additional analyses by amplicon sequence variant (ASV)-based clustering (SILVA 138.1 for alignment and classification) [64]. The number of quality reads was similar between OTU-based (average 28,610 ± 13,988 reads per sample; Table S1) and ASV-based (average 29,551 ± 19,502 reads per sample; Table S5a) approaches, resulting in nearly equivalent sequencing depth. Both approaches identified consistent taxonomic community structures at phylum and genus levels using Kruskal–Wallis testing with Benjamini–Hochberg FDR correction (*P*_adj_ > 0.05). Shared key taxa findings were such as elevated *Acinetobacter* and *Phyllobacterium* in active air samples, *Brevibacterium* in SW.C.OPD, and *Brachybacterium* in SW.C.PW (Figs 2A and B and Figs. S5 and S6). Alpha-diversity patterns were similar, with hospital N showing relatively higher diversity across methodologies (Figs. S4 and S6A to C) [65]. Beta-diversity analyses revealed statistically significant separation of microbiota by hospital location and department in both approaches (Fig. 3A and B and Fig. S7), suggesting that the observed microbial profiles reflected true environmental differences rather than artifacts of bioinformatic methodology. However, the ASV-based analysis exhibited reduced discriminatory power for departmental separation (Fig. S7) due to relatively lower sequencing coverage (Table S5B versus Table S2) [66], supporting the OTU-based approach as favorable for this dataset. Despite this, both pipelines confirmed the stability of core air microbiome signatures and helped validate the robustness of our ecological hospital microbiome conclusions.

Together, these findings demonstrated that hospital air microbiomes are shaped by several factors, including area, ventilation strategy, sampling method, and geographic location, all playing significant roles. By integrating taxonomic and inferred functional analyses, this study reported microbiome differences involved microbial metabolic potential differences across hospital locations and areas. The findings supported that hospital air is not microbiologically uniform but instead reflects complex interactions among environmental design, human activities, ventilations (e.g., windows, doors, air conditioners, and HEPA filters) and regional context. Improved understanding of these interactions could provide strategies to optimize hospital air quality, reduce the risk of HAIs, and protect patients, healthcare workers, and visitors [67–69]. Nonetheless, this study’s species-level identification is via the species-level assignments from the 16*S* rRNA V3 to V5 region, so additional validation for species, such as metagenomic sequencing or whole-genome sequencing, may be performed for validation and a seasonal effect between active air-pump sample (June to July) and passive air-grille swab and HEPA filter sample (November to December) collections. The additional methodological validations across various bioinformatic approaches (OTU and ASV) and statistical tests (parametric and nonparametric) enhanced the reliability and reproducibility of our reports regarding hospital air microbiome heterogeneity.

## Conclusion

This study characterized airborne bacterial microbiomes across hospital environments and sampling approaches in 2 hospitals located in a metropolitan and a smaller city in Thailand. The results demonstrated that airborne bacterial community composition and diversity varied significantly according to hospital location, functional area, and sampling method. Passive air-grille swab sampling captured higher microbial diversity and biomass than active air-pump sampling, while environments with mechanical filtration, particularly HEPA-filtered ORs, showed markedly reduced diversity compared with more open or highly occupied areas such as OPDs and Rs. Differences between hospitals further suggest that regional context and environmental conditions contribute to shaping airborne microbial communities. Predicted functional profiles also varied among sampling types and locations, indicating potential differences in microbial ecological functions within hospital air environments. Overall, these findings highlight the importance of environmental conditions and sampling strategies in characterizing hospital airborne microbiomes and provide a foundation for future studies investigating their potential implications for hospital environmental management.

## Materials and Methods

### Sample collections

Sampling sites were selected from 2 locations in Thailand, representing the metropolitan area (hospital C) and the smaller city area (hospital N). Sampling at hospital C included areas E, OPD, ORs, ORFs, PWs, and R. Sampling at hospital N included areas examination room (ER), E, OPD, OR, PW, and R.

Sampling methods included active air-pump and passive air-grille swab sampling using sterilized filters and swabs (except ORF that used HEPA filter as sample), respectively. All collected samples were transported at 4 °C and processed for genetic extraction upon arrival at the laboratory or stored at −80°C. Active air-pump sampling was collected during 0900 to 1700 h in 2021 July and 2022 June (as insufficient metagenomic DNA from 2021 July active air-pump sample collection, the samples were collected again around the similar season of the following year to prevent seasonal bias). The active air-pump apparatus utilized a mixed cellulose ester filter membrane of 0.2 μm in pore size and 47 mm in diameter and a flow rate pump of 100 l/min for 2 h, except for elevators using 100 l/min for 1 h. The apparatus was placed at a height of 1 m above the floor [70–72], and the apparatus was sterilized with 70% ethanol prior to each sampling. For passive air-grille swab sampling was collected using sterile cotton swabs soaked in a phosphate-buffered saline solution with 0.05% Tween 20. Each swab covered a 30 cm × 30 cm area, with triplicate swabs per sample and 3 to 5 independently replicates for each area [73], collected between 0900 and 1700 h in 2022 November to December. Triplicates were collected on the same day. The sampling timing, dates, and hours of filtration were identical for both hospitals.

### Metagenomic DNA extraction

The filters for active air-pump sampling, 3 independent filters per reaction, were placed in the 5-ml PowerWater DNA Bead Tube (QIAGEN, Hilden, Germany) [74] in solution PW1, and the swabs for passive air-grille or HEPA-filter swab sampling, 3 independent triplicates per reaction, were metagenomic extracted according to the DNeasy PowerWater Kit and DNeasy PowerSoil Kit (QIAGEN, Hilden, Germany) manufacturer’s protocols, respectively [74–76]. The quality and quantity of extracted metagenomic DNA were checked by agarose gel electrophoresis and nanodrop spectrophotometry [75,77]. Note that due to insufficient metagenomic concentration of active air-pump samples, all air-pump sample metagenomic DNA from the same hospital were pooled into 2 replicates (A.C.r1 and A.C.r2 for hospital C and A.N.r1 and A.N.r2 for hospital N).

### 16*S* rRNA gene library preparation and next generation sequencing

The metagenomic DNA were amplified the 16*S* rRNA gene hypervariable region V3 to V5 using universal primers 342F (5′-GGRGGCAGCAGTNGGGAA-3′) and 895R (5′-TGCGDCCGTACTCCCCA-3′) [78]. Each 25 μl of polymerase chain reaction (PCR) reaction comprised 1× EmeraldAmp GT PCR Master Mix (TakaRa Bio, Shiga, Japan), 0.3 μM of each primer, and 20 to 50 ng of the metagenomic DNA (or sterile water for negative control). The PCR thermocycling conditions were 94 °C for 3 min, 35 cycles of 94 °C for 45 s, 50 °C for 1 min, and 72°C for 1 min and 30 s, followed by 72 °C for 10 min [77,78]. The amplicons were prepared the 16*S* rRNA gene library preparation using MiSeq Reagent Kit v2 (2 × 250 base pairs [bp]) (Illumina, CA, USA). Briefly, the amplicons were appended 5′ Illumina adapter and unique barcode sequences for categorization of samples by PCR, then the ~700-bp amplicons were purified using PureDireX PCR Clean-Up & Gel Extraction Kit (GeneDireX, Keelung, Taiwan) and quantified by Qubit fluorometer (Invitrogen, Waltham, USA), and the libraries of different 16*S* rRNA amplicon samples were pooled into equimolar for sequencing [77]. Note that no amplicon for the PCR negative control. Sequencing was performed on MiSeq platform (Illumina) following the manufacturer’s protocols at Omics Science and Bioinformatics Center, Chulalongkorn University (Bangkok, Thailand).

### Bioinformatic and statistical analyses

Raw sequences were processed following Mothur 1.47.0’s standard operating procedures [79,80]. Quality screening included removal of (a) a short read length of <400 nucleotides excluding primer and barcode sequences, (b) ambiguous bases of >10 nucleotides, (c) chimera sequences, and (d) homopolymers of >10 nucleotides. The quality reads were aligned against SILVA 138.2 database to remove mitochondria, chloroplast and unknown lineages, and used Greengenes 13.8.99 database to classify into OTUs at phylum, class, order, family, genus, and specie levels. OTUs were clustered at 97% sequence similarity (distance cutoff, 0.03) [81]. Taxonomic assignments were performed using a bootstrap confidence threshold of 80% [82,83]; assignments with a confidence of <80% were classified as unassigned at that taxonomic level. Species-level identifications represented best-match assignments and should be interpreted as putative, as 16*S* rRNA V3 to V5 sequences provided limited resolution at the species level (65% to 83% accuracy) [29]. Genus-level classifications (>90% accuracy) [80] were considered more reliable and used for primary ecological interpretations. To ensure comparability across samples, all samples were normalized to equal sequencing depth of 12,041 quality reads per sample as the baseline normalization for calculating taxonomic relative abundances and prior to any statistical comparisons. Good’s coverage (sequencing depth relative to true sample diversity estimate) and alpha-diversity (number of OTUs, Chao richness, and Shannon diversity) were calculated using the standard pipeline in Mothur 1.47.0 [80]. Beta-diversity was computed using Morisita–Horn dissimilarity indices, and nonmetric multidimensional scaling (NMDS) along with representative species and Spearman’s correlation factors were computed using Mothur 1.47.0 [77,79,80]. The NMDS graph was generated with R Studio 4.3.1 and clustered at 90% confidence ellipse with multivariate normal distribution. The phylogenetic clustering was constructed with Jaccard dissimilarity indices. LEfSe for species biomarkers was analyzed following established protocols (*P* < 0.05) that utilized unrarefied sequences as input and the 16*S* rRNA gene copy number normalization step for percent compositions [77,84]. Estimates of the microbial metabolic profiles were determined by PICRUSt based on the reference genome annotations in KEGG [85] and statistically compared by STAMP (Statistical Analysis of Metagenomic Profiles) following established protocols [86]. Functional prediction accuracy was validated by weighted NSTI scores and the acceptable NSTI thresholds supported reliable functional characterization (Table S6). In addition to OTU clustering, ASVs were identified using the SILVA 138.1 to validate the consistency of the analysis pipelines: The classification was executed in Mothur with a bootstrap confidence cutoff of 80%, alpha- and beta-diversity analyses were performed on a subsampled dataset of 10,146 quality reads per sample (similar to OTU-based), and the relative abundance calculations were conducted using the nonsubsampled (total) dataset to preserve the complete taxonomic profile and minimize loss of rare taxa. The geographic coordinates (Global Positioning System) of hospitals were precisely identified using Google Maps and Google Earth Pro 7.3 [87]. Land-use data were retrieved from the OpenStreetMap database, and the percentage of green space was determined from dividing the total vegetation area by the total area of the 5-km buffer [88,89] (Table S7 displayed raw calculated land-use density data).

Statistical differences in alpha-diversity and relative abundance between samples (or groups) were assessed using Student’s *t* tests (or Mann–Whitney *U* test for nonparametric data; Fig. S4), and among samples (or groups) using ANOVA with Tukey’s honestly significantly different (HSD) test (or Kruskal–Wallis test with Dunn’s post hoc test for nonparametric data; Fig. S5) (*P* < 0.05) [77,80]. The *P* values were adjusted using the Benjamini–Hochberg FDR correction (*P*_adj_ < 0.05). Beta-diversity was assessed by AMOVA (Mothur 1.47.0) on Morisita–Horn dissimilarity matrices. Homogeneity of dispersions was verified using PERMDISP (vegan 2.6-4 in R 4.3.2) [90]; all swab samples showed equal dispersion (*P* > 0.05; Table S8), validating AMOVA results. For functional prediction statistics by STAMP, Welch’s *t* test and ANOVA with FDR correction (data treated as proportions) was utilized. LEfSe used nonparametric tests (Kruskal–Wallis and Wilcoxon rank sum tests) on relative abundances, and statistical significance was reported as logarithmic LDA scores (*P* < 0.05, LDA > 3.0).

## Data Availability

Nucleic acid sequences from this study are available in the Sequence Read Archive database of National Center for Biotechnology Information under accession number PRJNA1272103.
