# Supplementary material for: Air Bacterial Microbiomes in Hospitals: Case Studies from a Metropolis and a Small City of Thailand
Source: Comput Struct Biotechnol J. 2026 Apr 29;35(1):0068. doi: 10.34133/csbj.0068 (PMC13125743; doi:10.34133/csbj.0068)
Supplement: Supplementary 1 — Figs. S1 to S7 Tables S1 to S8 [file csbj.0068.f1.zip › csbj.0068.f1.docx]

**Supporting information**


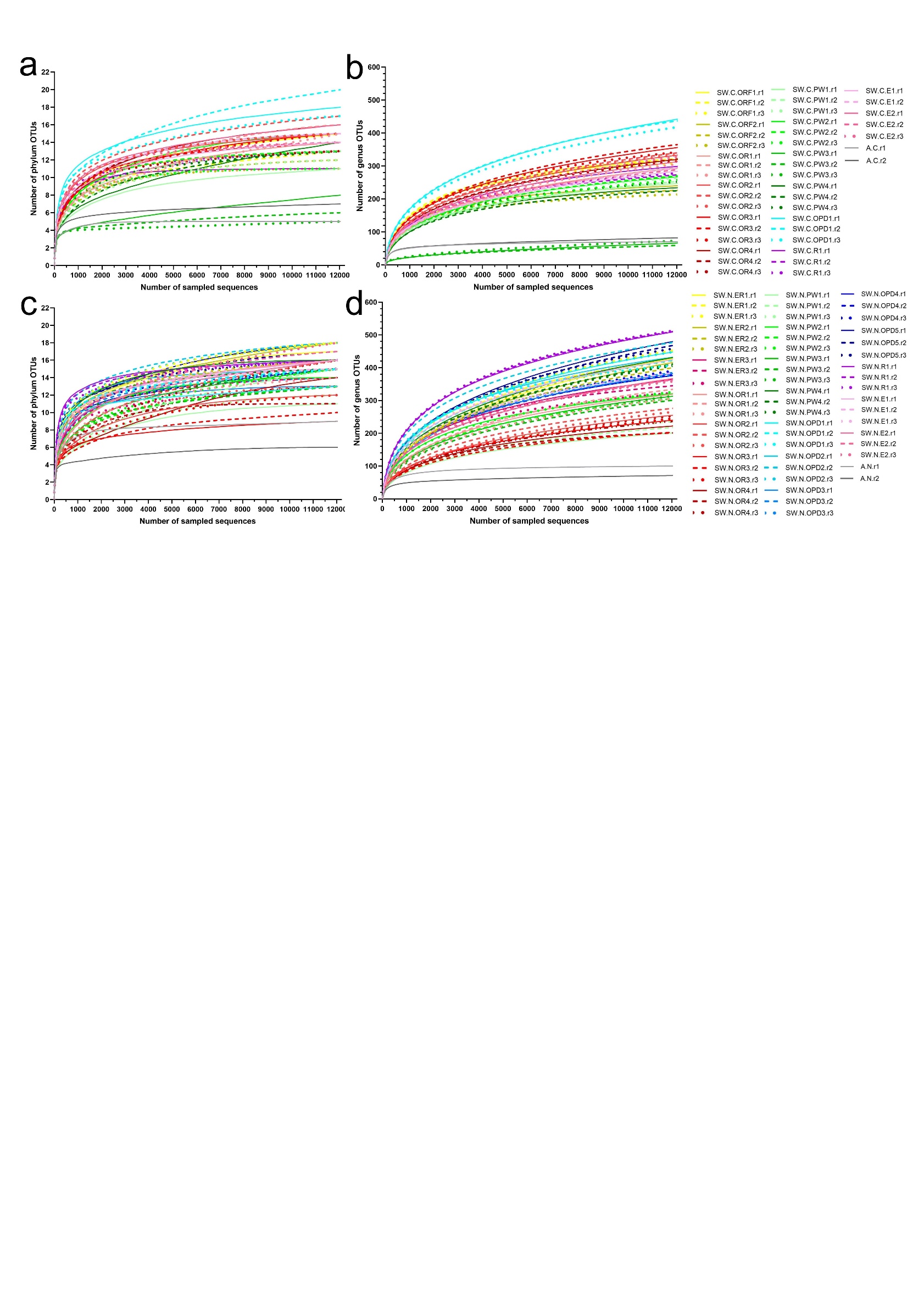


**Fig. S1.** Rarefaction curves of OTUs at levels phylum (**a** and **c**) and genus (**b** and **d**), of hospitals C and N, respectively. Sample IDs were abbreviated as follows: sampling method (SW or A), followed by hospital (C or N), area (e.g., OR, PW, ER), and the replicate number.

**
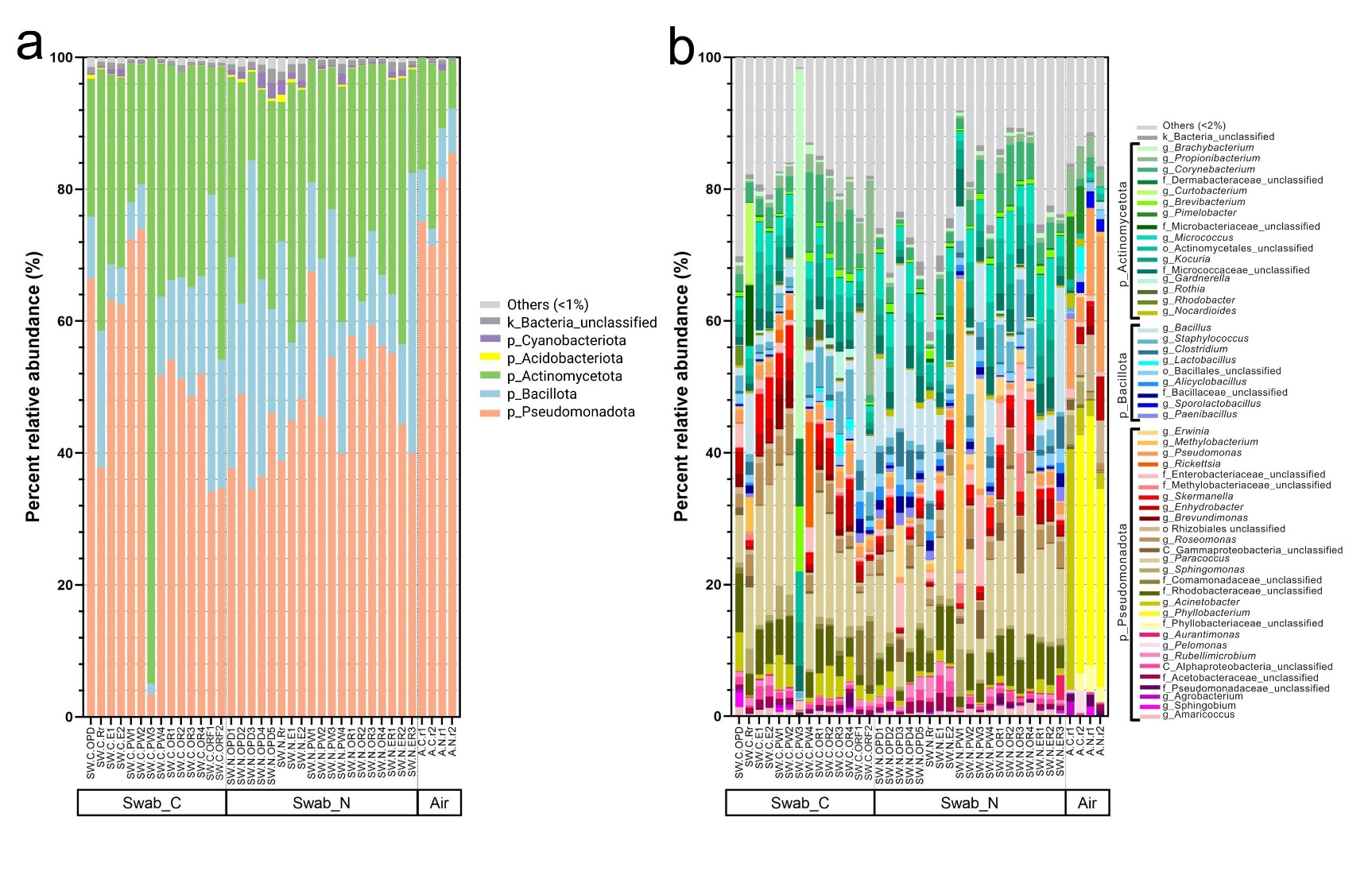
**

**Fig. S2.** Percent relative compositions of individual samples in (**a**) phylum and (**b**) genus levels.

k_ abbreviates kingdom; p_, phylum; c_, class; o_, order; f_, family; g_, genus; and Others,

phyla (or genera) with < 1% (or 2%).

**
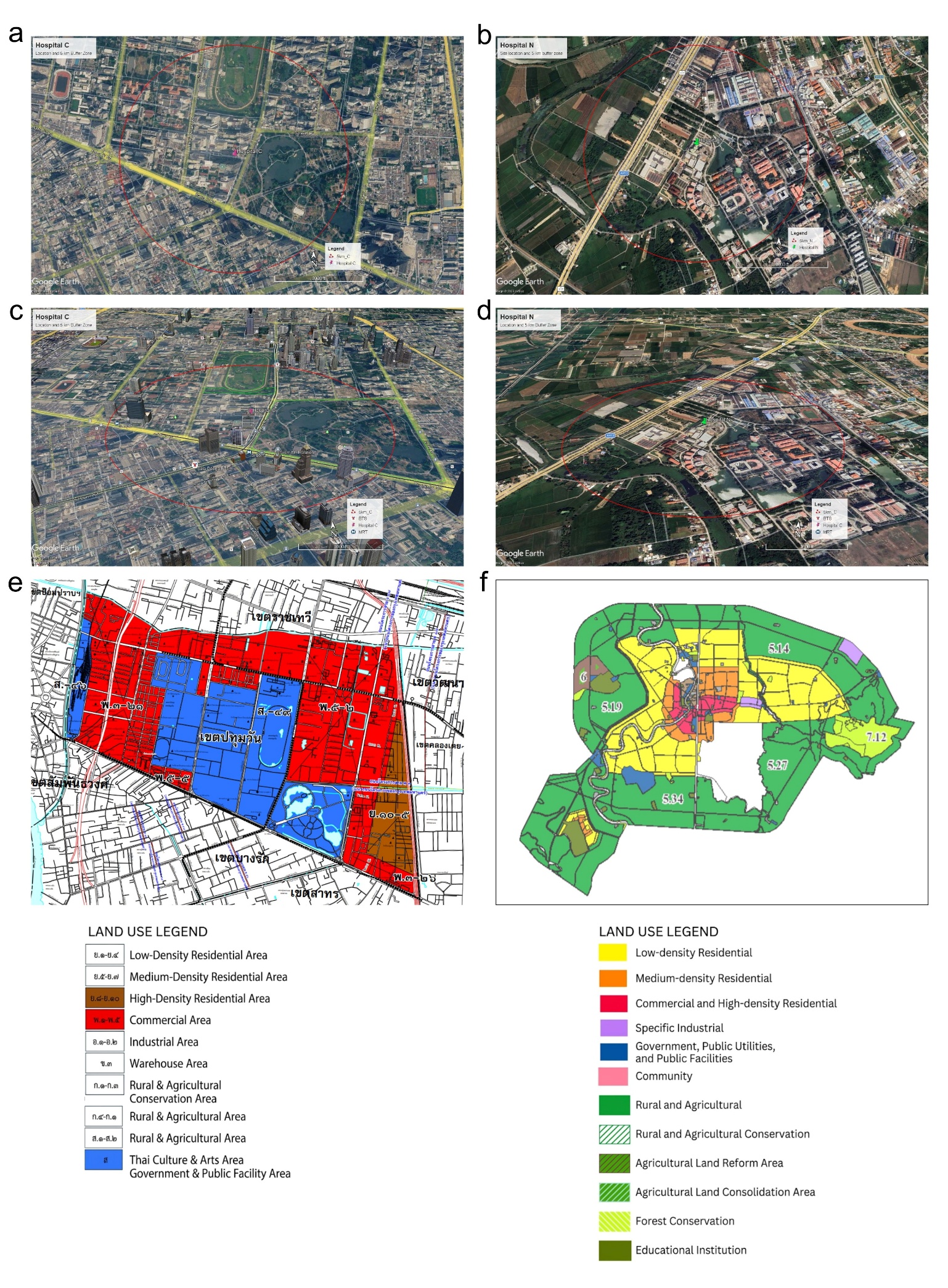
**

**Fig. S3.** Geographic and zoning profiles of study sites. (**a** and **b**) Satellite images showing 5-km radius (red circles) around hospitals C (Bangkok) and N (Phitsanulok), (**c** and **d**) three dimension perspective views illustrating more dense of high-rise developments and mass transits (Bangkok Mass Transit System, BTS and Metropolitan Rapid Transit, MRT) around hospital C versus low-rise fragmented developments for hospital N, and (**e** and **f**) official zoning maps (Department of Public Works, Town and Country Plannings) for Hospital C in commercial (red) zone (Pathum Wan District, Bangkok) versus Hospital N in agricultural (green) zone (Mueang District, Phitsanulok).

**
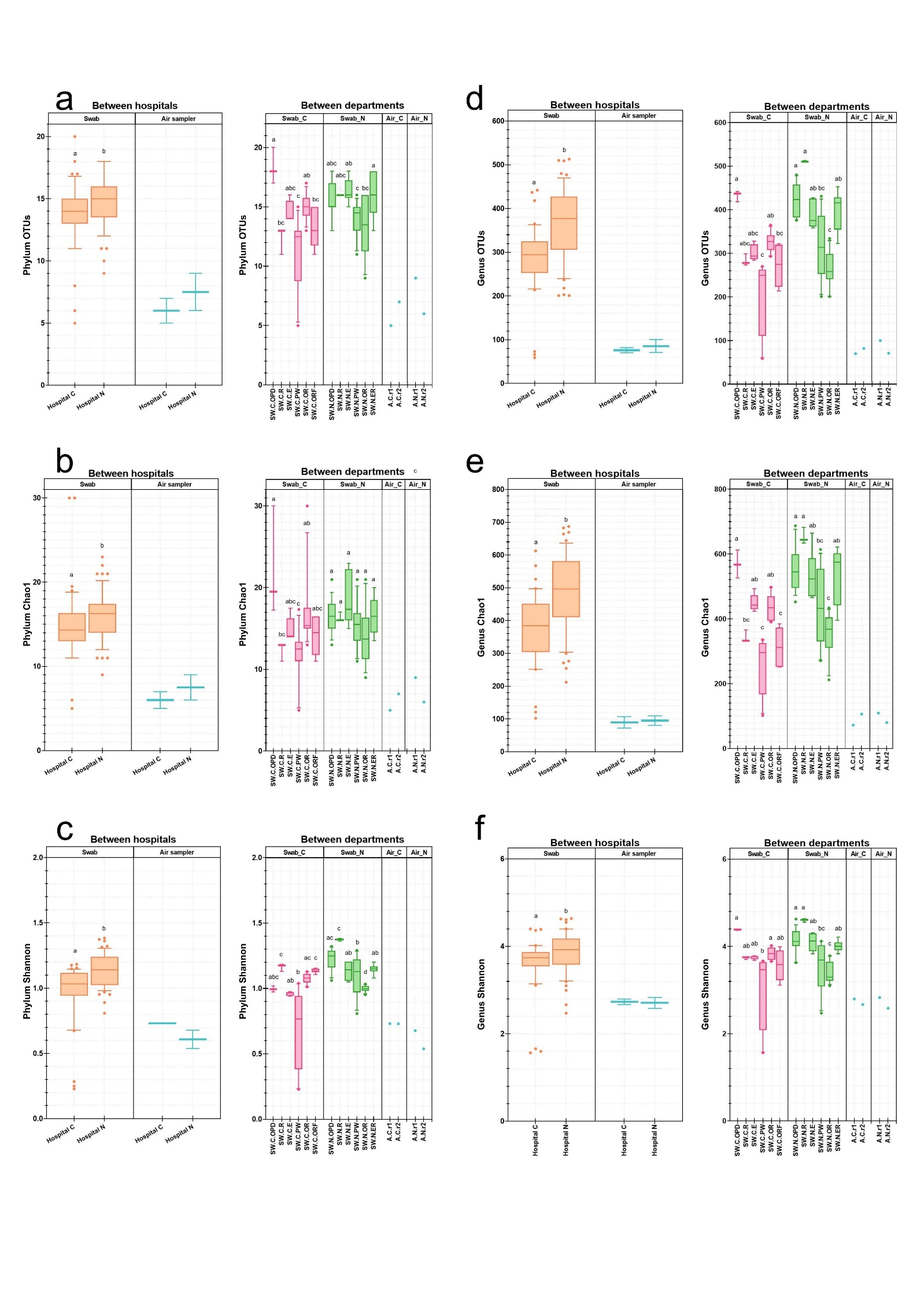
**

**Fig. S4.** Non-parametric statistical computations of alpha diversity indices at phylum (**a–c**: OTUs, Chao1 and Shannon) and genus (**d–f**: OTUs, Chao1 and Shannon) levels, between hospitals using Mann–Whitney U test, and among different departments using Kruskal–Wallis test with Dunn’s post-hoc analysis and Benjamini–Hochberg FDR correction. Different alphabet letters represent statistically significant *P*_adj_ < 0.05.

**
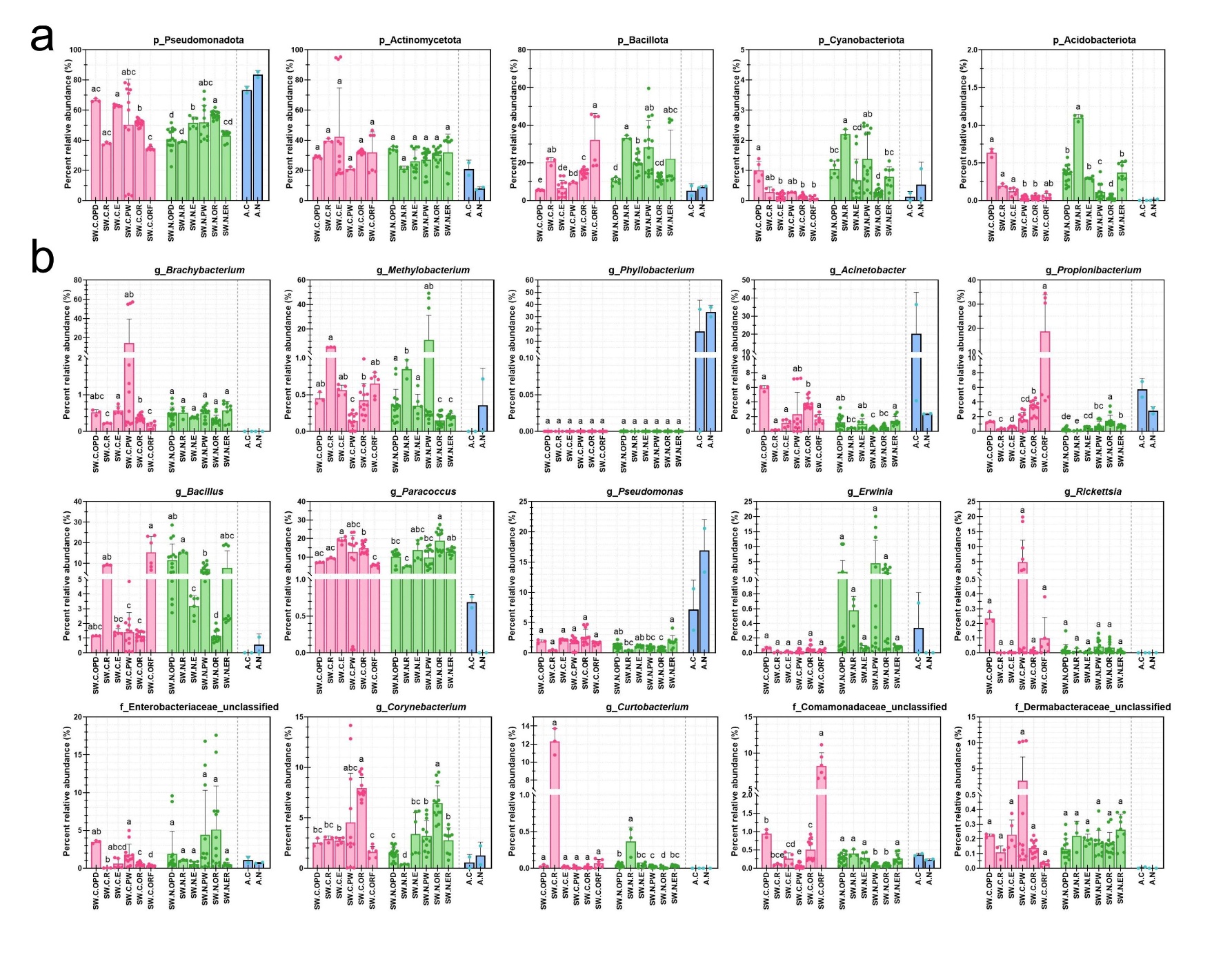
**

**Fig. S5.** Non-parametric statistical computations for the relative abundances of (**a**) top-5 phyla and (**b**) top-15 genera, using Kruskal–Wallis with Dunn’s post-hoc analysis and Benjamini–Hochberg FDR correction. Different alphabet letters represent statistically significant *P*_adj_ < 0.05.

**
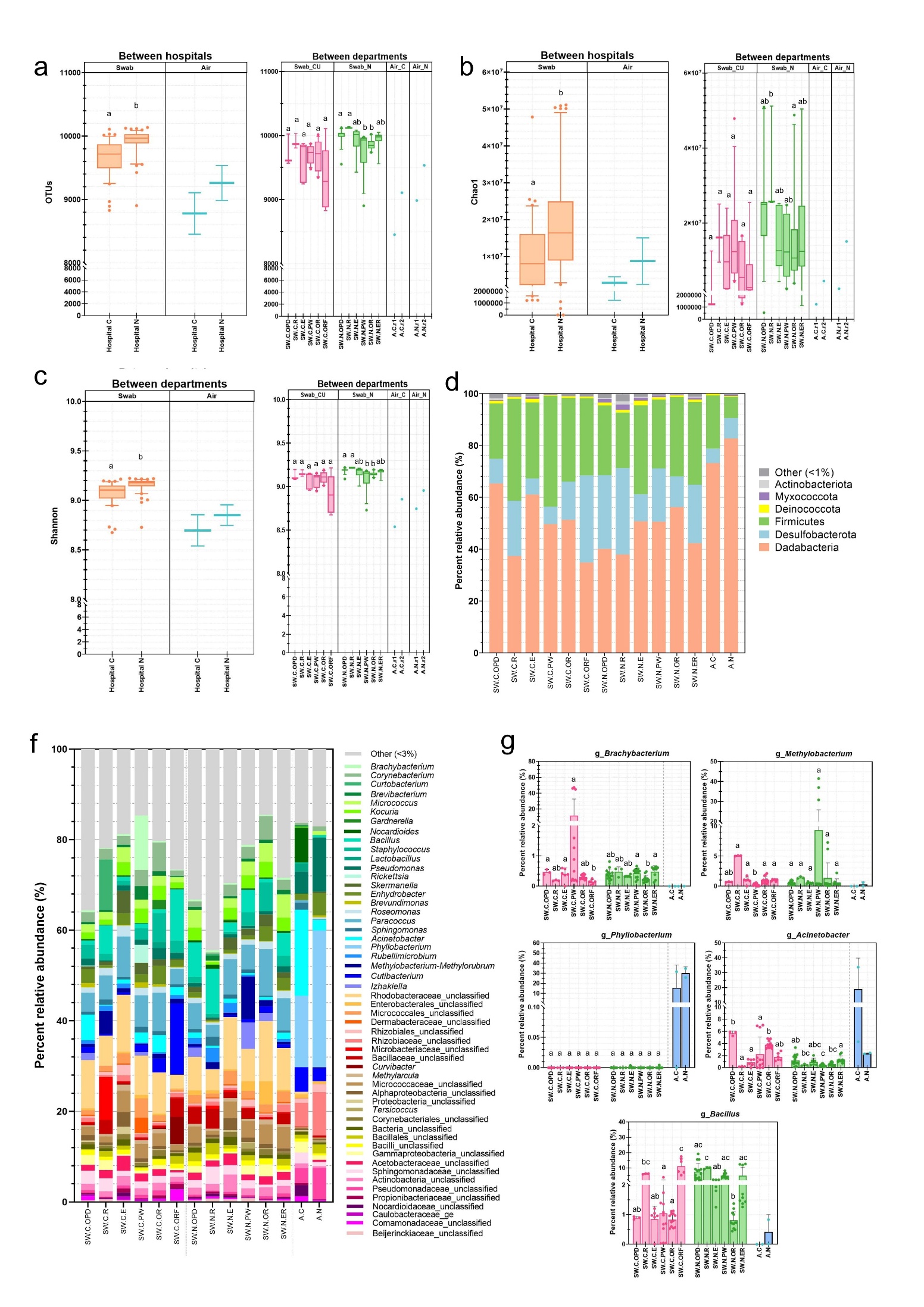
**

**Fig. S6.** Microbial alpha diversity and percent composition based on ASV pipeline results. **(a)** Observed OTUs, **(b)** Chao1, **(c)** Shannon, **(d)** average percent phylum composition, **(f)** average percent genus compositions, and **(g)** statistical comparisons of 5 predominated genera (comparisons between hospitals using Mann–Whitney U test, and among departments using Kruskal–Wallis with Dunn’s post-hoc analysis and Benjamini–Hochberg FDR correction. Different alphabet letters represent statistically significant *P*_adj_ < 0.05. Error bars represent the standard error of the mean.

**
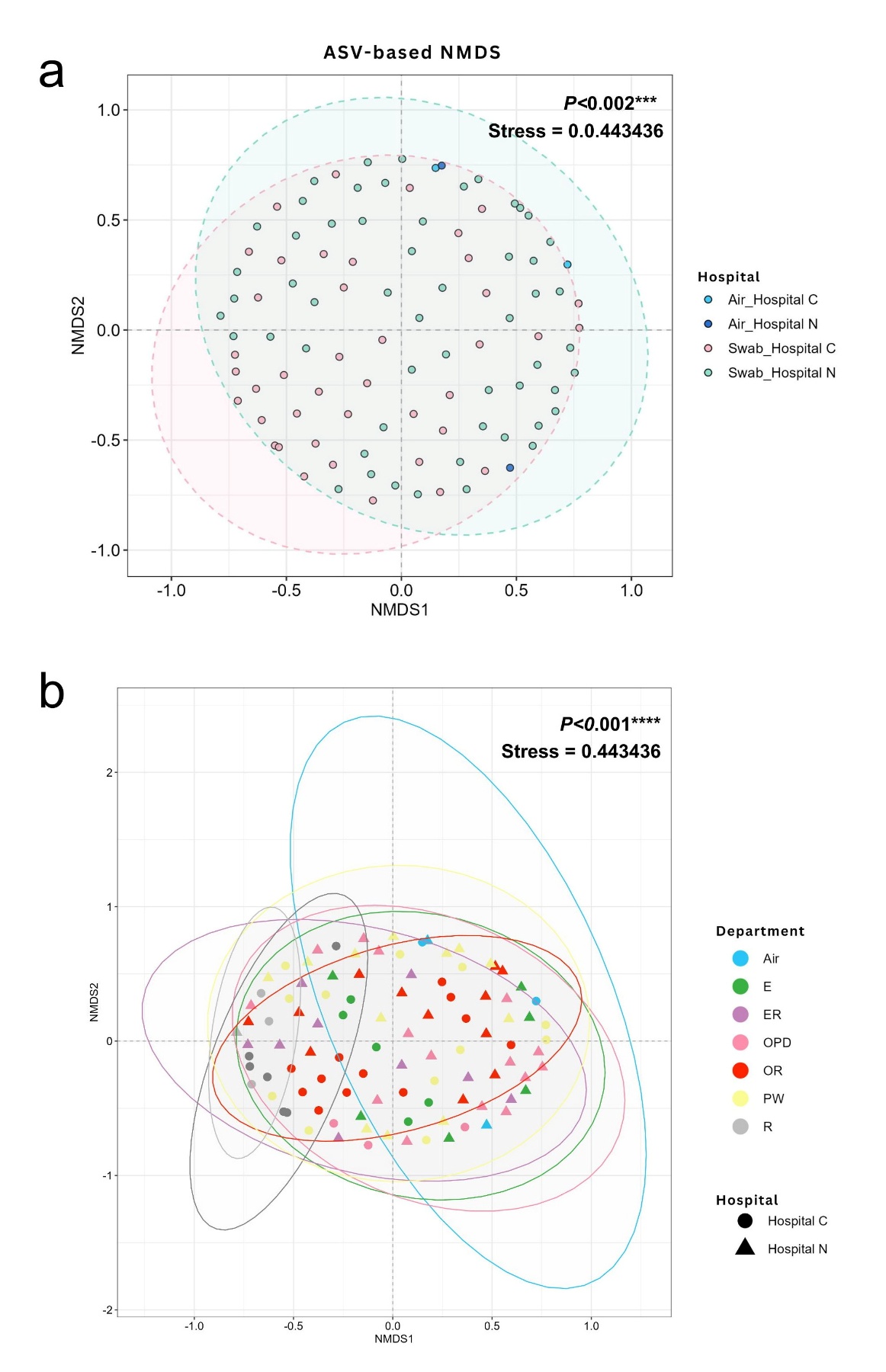
**

**Fig. S7.** NMDS (by Morisita-Horn dissimilarity indices) of ASV pipeline results. **(a)** between hospitals C and N, and **(b)** among different hospital departments. Ellipses represent 90% confidence clusters from multivariate normal distribution. Statistical differences were calculated by AMOVA (* infers *P* < 0.05; **, *P* < 0.01; ***, *P* < 0.005, and ****, *P* < 0.001).

**Table S1.** Sample IDs, along metagenomic DNA concentrations and number of quality reads. Sample abbreviations (sample IDs) are as follows: active air-pump samples or passive air-grille-swab samples (abbreviated A or SW) followed by hospital (King Chulalongkorn Memorial Hospital, C; or Naresuan University Hospital, N), areas (elevator, E; outpatient department, OPD; operating rooms, OR; HEPA-filter in operating rooms, ORF; patient wards, PW; restaurants, R; examination room, ER), and the replicate number.

| **Sample IDs** | **DNA concentration (ng/m^3^)** | **Raw reads** | **Quality reads** |
| --- | --- | --- | --- |
| A.C.r1 | 1.18 | 149,520 | 47,589 |
| A.C.r2 | 1.029 | 68,831 | 22,209 |
| A.N.r1 | 6.315 | 110,867 | 36,673 |
| A.N.r2 | 7.704 | 90,275 | 17,913 |
| **Sample IDs** | **DNA concentration (ng/m^2^)** | **Raw reads** | **Quality reads** |
| SW.C.OPD1.r1 | 233.333 | 79,592 | 53,498 |
| SW.C.OPD1.r2 | 165.925 | 77,729 | 52,007 |
| SW.C.OPD1.r3 | 222.962 | 40,465 | 24,555 |
| SW.C.R.r1 | 243.703 | 44,573 | 24,471 |
| SW.C.R.r2 | 285.185 | 42,105 | 25,106 |
| SW.C.R.r3 | 259.259 | 46,456 | 24,099 |
| SW.C.E1.r1 | 160.74 | 68,733 | 50,130 |
| SW.C.E1.r2 | 233.333 | 44,729 | 32,026 |
| SW.C.E2.r1 | 311.111 | 41,102 | 26,767 |
| SW.C.E2.r2 | 212.592 | 42,913 | 28,087 |
| SW.C.E2.r3 | 134.814 | 84,803 | 59,990 |
| SW.C.PW1.r1 | 342.222 | 48,622 | 28,716 |
| SW.C.PW1.r2 | 316.296 | 44,875 | 29,495 |
| SW.C.PW1.r3 | 331.851 | 37,558 | 23,413 |
| SW.C.PW2.r1 | 425.185 | 37,950 | 24,976 |
| SW.C.PW2.r2 | 461.481 | 31,179 | 20,927 |
| SW.C.PW2.r3 | 487.407 | 46,972 | 31,133 |
| SW.C.PW3.r1 | 508.148 | 28,236 | 21,617 |
| SW.C.PW3.r2 | 440.74 | 29,855 | 22,666 |
| SW.C.PW3.r3 | 622.222 | 32,388 | 24,611 |
| SW.C.PW4.r1 | 674.074 | 40,726 | 22,309 |
| SW.C.PW4.r2 | 715.555 | 45,407 | 26,616 |
| SW.C.PW4.r3 | 725.925 | 38,078 | 23,516 |
| SW.C.OR1.r1 | 575.555 | 36,741 | 24,132 |
| SW.C.OR1.r2 | 622.222 | 34,524 | 23,365 |
| SW.C.OR1.r3 | 663.703 | 47,953 | 28,266 |
| SW.C.OR2.r1 | 67.407 | 42,757 | 26,739 |
| SW.C.OR2.r2 | 36.296 | 40,840 | 24,941 |
| SW.C.OR2.r3 | 93.333 | 43,368 | 26,704 |
| SW.C.OR3.r1 | 160.74 | 64,982 | 40,958 |
| SW.C.OR3.r2 | 233.333 | 78,952 | 51,095 |
| SW.C.OR3.r3 | 254.074 | 73,973 | 40,700 |
| SW.C.OR4.r1 | 140 | 82,392 | 45,897 |
| SW.C.OR4.r2 | 254.074 | 94,889 | 59,733 |
| SW.C.OR4.r3 | 165.925 | 96,689 | 56,119 |
| SW.C.ORF1.r1 | 637.777 | 42,468 | 15,448 |
| SW.C.ORF1.r2 | 679.259 | 94,664 | 32,289 |
| SW.C.ORF1.r3 | 617.037 | 118,355 | 30,268 |
| SW.C.ORF2.r1 | 176.296 | 100,182 | 38,213 |
| SW.C.ORF2.r2 | 165.925 | 90,136 | 30,235 |
| SW.C.ORF2.r3 | 129.629 | 67,295 | 28,305 |
| SW.N.OPD1.r1 | 222.962 | 46,883 | 26,145 |
| SW.N.OPD1.r2 | 264.444 | 34,151 | 19,424 |
| SW.N.OPD1.r3 | 357.777 | 33,606 | 16,665 |
| SW.N.OPD2.r1 | 404.444 | 25,255 | 16,392 |
| SW.N.OPD2.r2 | 373.333 | 232,881 | 72,802 |
| SW.N.OPD2.r3 | 425.185 | 28,914 | 22,702 |
| SW.N.OPD3.r1 | 575.555 | 35,421 | 22,315 |
| SW.N.OPD3.r2 | 617.037 | 40,962 | 22,288 |
| SW.N.OPD3.r3 | 549.629 | 32,406 | 15,752 |
| SW.N.OPD4.r1 | 119.259 | 44,133 | 17,672 |
| SW.N.OPD4.r2 | 243.703 | 59,181 | 24,900 |
| SW.N.OPD4.r3 | 77.777 | 65,973 | 22,116 |
| SW.N.OPD5.r1 | 694.814 | 35,838 | 25,649 |
| SW.N.OPD5.r2 | 689.629 | 42,621 | 31,402 |
| SW.N.OPD5.r3 | 420 | 37,699 | 26,623 |
| SW.N.R.r1 | 124.444 | 45,126 | 16,520 |
| SW.N.R.r2 | 197.037 | 48,453 | 15,758 |
| SW.N.R.r3 | 233.333 | 53,166 | 17,032 |
| SW.N.E1.r1 | 585.925 | 46,547 | 26,529 |
| SW.N.E1.r2 | 466.666 | 30,129 | 12,041 |
| SW.N.E1.r3 | 202.222 | 53,835 | 27,808 |
| SW.N.E2.r1 | 1083.703 | 39,409 | 20,444 |
| SW.N.E2.r2 | 295.555 | 47,065 | 20,967 |
| SW.N.E2.r3 | 1726.666 | 147,836 | 27,857 |
| SW.N.PW1.r1 | 274.814 | 39,843 | 22,553 |
| SW.N.PW1.r2 | 368.148 | 90,223 | 55,025 |
| SW.N.PW1.r3 | 471.851 | 50,800 | 25,991 |
| SW.N.PW2.r1 | 435.555 | 51,195 | 23,723 |
| SW.N.PW2.r2 | 456.296 | 55,831 | 24,658 |
| SW.N.PW2.r3 | 502.962 | 41,310 | 23,201 |
| SW.N.PW3.r1 | 534.074 | 72,172 | 19,628 |
| SW.N.PW3.r2 | 585.925 | 72,446 | 16,999 |
| SW.N.PW3.r3 | 471.851 | 47,255 | 23,527 |
| SW.N.PW4.r1 | 492.592 | 30,891 | 21,735 |
| SW.N.PW4.r2 | 492.592 | 37,977 | 26,694 |
| SW.N.PW4.r3 | 207.407 | 82,144 | 47,885 |
| SW.N.OR1.r1 | 383.703 | 59,848 | 18,582 |
| SW.N.OR1.r2 | 466.666 | 230,472 | 47,670 |
| SW.N.OR1.r3 | 321.481 | 47,341 | 21,260 |
| SW.N.OR2.r1 | 155.555 | 72,315 | 18,783 |
| SW.N.OR2.r2 | 145.185 | 38,374 | 27,650 |
| SW.N.OR2.r3 | 114.074 | 51,436 | 20,305 |
| SW.N.OR3.r1 | 305.925 | 62,543 | 19,084 |
| SW.N.OR3.r2 | 404.444 | 82,402 | 9,841 |
| SW.N.OR3.r3 | 378.518 | 259,015 | 52,864 |
| SW.N.OR4.r1 | 222.962 | 67,040 | 10,162 |
| SW.N.OR4.r2 | 171.111 | 51,639 | 7,703 |
| SW.N.OR4.r3 | 212.592 | 57,926 | 28,983 |
| SW.N.ER1.r1 | 103.703 | 125,460 | 100,221 |
| SW.N.ER1.r2 | 88.148 | 42,178 | 26,221 |
| SW.N.ER1.r3 | 165.925 | 41,332 | 23,899 |
| SW.N.ER2.r1 | 124.444 | 37,879 | 24,187 |
| SW.N.ER2.r2 | 88.148 | 64,835 | 19,570 |
| SW.N.ER2.r3 | 191.851 | 45,262 | 25,433 |
| SW.N.ER3.r1 | 103.703 | 48,296 | 26,200 |
| SW.N.ER3.r2 | 77.777 | 38,551 | 19,410 |
| SW.N.ER3.r3 | 25.925 | 40,905 | 22,214 |

**Table S2.** Good’s coverage and alpha diversity indices at (**a**) phylum and (**b**) genus levels.

(**a**)

| **Sample IDs** | **Good's coverage** | **No. of OTUs** | **Chao1** | **Shannon** |
| --- | --- | --- | --- | --- |
| A.C.r1 | 1 | 5 | 5 | 0.730658 |
| A.C.r2 | 0.999917 | 7 | 7 | 0.730082 |
| A.N.r1 | 0.999917 | 9 | 9 | 0.677548 |
| A.N.r2 | 1 | 6 | 6 | 0.538413 |
| SW.C.ORF1.r1 | 0.999751 | 15 | 16.5 | 1.148734 |
| SW.C.ORF1.r2 | 0.999751 | 13 | 16 | 1.132161 |
| SW.C.ORF1.r3 | 0.999751 | 15 | 16.5 | 1.129945 |
| SW.C.ORF2.r1 | 0.999917 | 13 | 13 | 1.155075 |
| SW.C.ORF2.r2 | 0.999917 | 12 | 12 | 1.104501 |
| SW.C.ORF2.r3 | 1 | 11 | 11 | 1.13147 |
| SW.C.E1.r1 | 0.999917 | 14 | 14 | 0.943676 |
| SW.C.E1.r2 | 0.999917 | 15 | 15 | 0.941982 |
| SW.C.E2.r1 | 0.999751 | 16 | 17.5 | 0.974908 |
| SW.C.E2.r2 | 0.999917 | 14 | 14 | 0.968383 |
| SW.C.E2.r3 | 0.999917 | 14 | 14 | 0.965567 |
| SW.C.OPD1.r1 | 0.999751 | 18 | 19.5 | 1.020331 |
| SW.C.OPD1.r2 | 0.999585 | 20 | 30 | 0.973729 |
| SW.C.OPD1.r3 | 0.999834 | 17 | 17.25 | 0.99409 |
| SW.C.OR1.r1 | 0.999834 | 14 | 14.333333 | 1.041474 |
| SW.C.OR1.r2 | 0.999834 | 16 | 16.166667 | 1.011883 |
| SW.C.OR1.r3 | 0.999502 | 15 | 30 | 1.024917 |
| SW.C.OR2.r1 | 0.999834 | 15 | 15.5 | 1.108827 |
| SW.C.OR2.r2 | 0.999751 | 17 | 18 | 1.122149 |
| SW.C.OR2.r3 | 0.999917 | 15 | 15 | 1.128768 |
| SW.C.OR3.r1 | 0.999834 | 15 | 15.25 | 1.098603 |
| SW.C.OR3.r2 | 0.999834 | 14 | 15 | 1.080532 |
| SW.C.OR3.r3 | 0.999751 | 15 | 16 | 1.111748 |
| SW.C.OR4.r1 | 0.999751 | 16 | 19 | 1.064646 |
| SW.C.OR4.r2 | 0.999917 | 15 | 15 | 1.077045 |
| SW.C.OR4.r3 | 0.999917 | 13 | 13 | 1.059571 |
| SW.C.PW1.r1 | 0.999834 | 11 | 12 | 0.738161 |
| SW.C.PW1.r2 | 0.999917 | 13 | 13 | 0.673564 |
| SW.C.PW1.r3 | 0.999917 | 12 | 12 | 0.89032 |
| SW.C.PW2.r1 | 0.999917 | 15 | 15 | 0.794168 |
| SW.C.PW2.r2 | 0.999917 | 13 | 13 | 0.843322 |
| SW.C.PW2.r3 | 1 | 11 | 11 | 0.693489 |
| SW.C.PW3.r1 | 0.999751 | 8 | 11 | 0.228071 |
| SW.C.PW3.r2 | 0.999917 | 6 | 6 | 0.250268 |
| SW.C.PW3.r3 | 0.999917 | 5 | 5 | 0.284135 |
| SW.C.PW4.r1 | 0.999585 | 14 | 17.333333 | 1.039608 |
| SW.C.PW4.r2 | 0.999917 | 13 | 13 | 1.033315 |
| SW.C.PW4.r3 | 0.999834 | 13 | 13.5 | 0.95802 |
| SW.C.R.r1 | 1 | 11 | 11 | 1.130668 |
| SW.C.R.r2 | 0.999917 | 13 | 13 | 1.175836 |
| SW.C.R.r3 | 0.999917 | 13 | 13 | 1.183476 |
| SW.N.E1.r1 | 0.999751 | 16 | 17.5 | 1.203978 |
| SW.N.E1.r2 | 0.999917 | 15 | 15 | 1.165375 |
| SW.N.E1.r3 | 0.999834 | 16 | 16.333333 | 1.204115 |
| SW.N.E2.r1 | 0.999668 | 16 | 22 | 1.050139 |
| SW.N.E2.r2 | 0.999834 | 17 | 17.2 | 1.119687 |
| SW.N.E2.r3 | 0.999585 | 18 | 23 | 1.060918 |
| SW.N.ER1.r1 | 0.999917 | 17 | 17 | 1.137464 |
| SW.N.ER1.r2 | 0.999751 | 18 | 18.75 | 1.173052 |
| SW.N.ER1.r3 | 0.999834 | 18 | 18.25 | 1.201209 |
| SW.N.ER2.r1 | 0.999668 | 18 | 20 | 1.162224 |
| SW.N.ER2.r2 | 0.999834 | 16 | 16.333333 | 1.124301 |
| SW.N.ER2.r3 | 0.999834 | 16 | 16.5 | 1.15476 |
| SW.N.ER3.r1 | 0.999917 | 15 | 15 | 1.149893 |
| SW.N.ER3.r2 | 0.999917 | 14 | 14 | 1.141112 |
| SW.N.ER3.r3 | 0.999834 | 13 | 13.5 | 1.077827 |
| SW.N.OPD1.r1 | 0.999834 | 13 | 14 | 1.244154 |
| SW.N.OPD1.r2 | 0.999834 | 15 | 15.5 | 1.248937 |
| SW.N.OPD1.r3 | 0.999751 | 15 | 18 | 1.248019 |
| SW.N.OPD2.r1 | 0.999917 | 15 | 15 | 1.191493 |
| SW.N.OPD2.r2 | 0.999834 | 18 | 18.333333 | 1.210391 |
| SW.N.OPD2.r3 | 0.999751 | 18 | 21 | 1.160486 |
| SW.N.OPD3.r1 | 1 | 13 | 13 | 1.096087 |
| SW.N.OPD3.r2 | 0.999917 | 14 | 14 | 1.112398 |
| SW.N.OPD3.r3 | 0.999751 | 15 | 16.5 | 1.061461 |
| SW.N.OPD4.r1 | 0.999751 | 15 | 16.5 | 1.279346 |
| SW.N.OPD4.r2 | 0.999751 | 15 | 16.5 | 1.321928 |
| SW.N.OPD4.r3 | 0.999834 | 15 | 15.5 | 1.288049 |
| SW.N.OPD5.r1 | 0.999834 | 18 | 18.2 | 1.303944 |
| SW.N.OPD5.r2 | 0.999834 | 17 | 17.333333 | 1.314761 |
| SW.N.OPD5.r3 | 0.999834 | 16 | 17 | 1.286591 |
| SW.N.OR1.r1 | 0.999834 | 16 | 16.25 | 1.033718 |
| SW.N.OR1.r2 | 0.999834 | 16 | 16.333333 | 1.015927 |
| SW.N.OR1.r3 | 0.999834 | 14 | 14.5 | 1.018992 |
| SW.N.OR2.r1 | 0.999917 | 12 | 12 | 0.95184 |
| SW.N.OR2.r2 | 0.999585 | 16 | 21 | 0.989813 |
| SW.N.OR2.r3 | 0.999917 | 13 | 13 | 1.008029 |
| SW.N.OR3.r1 | 0.999917 | 9 | 9 | 0.982511 |
| SW.N.OR3.r2 | 0.999834 | 10 | 11 | 0.987962 |
| SW.N.OR3.r3 | 0.999834 | 12 | 12.5 | 1.006011 |
| SW.N.OR4.r1 | 0.999751 | 14 | 14.428571 | 0.983006 |
| SW.N.OR4.r2 | 1 | 11 | 11 | 0.969442 |
| SW.N.OR4.r3 | 0.999585 | 16 | 19.333333 | 1.021987 |
| SW.N.PW1.r1 | 0.999917 | 11 | 11 | 0.808043 |
| SW.N.PW1.r2 | 0.999585 | 15 | 18.333333 | 0.889819 |
| SW.N.PW1.r3 | 0.999917 | 12 | 12 | 0.95269 |
| SW.N.PW2.r1 | 1 | 14 | 14 | 1.191479 |
| SW.N.PW2.r2 | 0.999834 | 13 | 13.5 | 1.123696 |
| SW.N.PW2.r3 | 0.999668 | 15 | 17 | 1.150269 |
| SW.N.PW3.r1 | 0.999668 | 15 | 21 | 1.133789 |
| SW.N.PW3.r2 | 0.999751 | 13 | 16 | 1.078784 |
| SW.N.PW3.r3 | 0.999834 | 13 | 13.5 | 1.022748 |
| SW.N.PW4.r1 | 1 | 16 | 16 | 1.29129 |
| SW.N.PW4.r2 | 0.999917 | 15 | 15 | 1.266396 |
| SW.N.PW4.r3 | 0.999751 | 15 | 16.5 | 1.234125 |
| SW.N.R.r1 | 0.999917 | 16 | 16 | 1.383591 |
| SW.N.R.r2 | 0.999917 | 16 | 16 | 1.360757 |
| SW.N.R.r3 | 0.999834 | 16 | 17 | 1.373827 |

(**b**)

| **Sample IDs** | **Good's coverage** | **No. of OTUs** | **Chao1** | **Shannon** |
| --- | --- | --- | --- | --- |
| A.C.r1 | 0.999585 | 70 | 72.5 | 2.796326 |
| A.C.r2 | 0.998422 | 82 | 106.428571 | 2.667347 |
| A.N.r1 | 0.999336 | 100 | 109.333333 | 2.829222 |
| A.N.r2 | 0.999086 | 71 | 80.166667 | 2.582187 |
| SW.C.ORF1.r1 | 0.994187 | 319 | 384.27027 | 3.993493 |
| SW.C.ORF1.r2 | 0.994851 | 322 | 369.275 | 3.881245 |
| SW.C.ORF1.r3 | 0.995183 | 309 | 359.090909 | 3.831931 |
| SW.C.ORF2.r1 | 0.996927 | 240 | 263.785714 | 3.258428 |
| SW.C.ORF2.r2 | 0.997259 | 226 | 252.4 | 3.113431 |
| SW.C.ORF2.r3 | 0.99701 | 214 | 251.058824 | 3.331776 |
| SW.C.E1.r1 | 0.99211 | 284 | 423.53125 | 3.772898 |
| SW.C.E1.r2 | 0.991114 | 294 | 451.527778 | 3.682953 |
| SW.C.E2.r1 | 0.991197 | 313 | 436.666667 | 3.769203 |
| SW.C.E2.r2 | 0.992276 | 289 | 441.785714 | 3.785683 |
| SW.C.E2.r3 | 0.989951 | 328 | 493 | 3.738104 |
| SW.C.OPD1.r1 | 0.987875 | 442 | 612.725806 | 4.389036 |
| SW.C.OPD1.r2 | 0.989785 | 437 | 526.321429 | 4.397961 |
| SW.C.OPD1.r3 | 0.989121 | 418 | 567.385965 | 4.364718 |
| SW.C.OR1.r1 | 0.992027 | 295 | 418.243243 | 3.656885 |
| SW.C.OR1.r2 | 0.992276 | 293 | 392.488372 | 3.661324 |
| SW.C.OR1.r3 | 0.992193 | 305 | 392.42 | 3.656074 |
| SW.C.OR2.r1 | 0.991197 | 333 | 456.666667 | 3.791836 |
| SW.C.OR2.r2 | 0.990865 | 340 | 497.763158 | 3.816925 |
| SW.C.OR2.r3 | 0.99128 | 334 | 474 | 3.802839 |
| SW.C.OR3.r1 | 0.990532 | 356 | 496.021739 | 3.997371 |
| SW.C.OR3.r2 | 0.991031 | 365 | 453.892308 | 4.01961 |
| SW.C.OR3.r3 | 0.992609 | 342 | 413.2 | 4.007932 |
| SW.C.OR4.r1 | 0.99211 | 320 | 398.333333 | 3.89791 |
| SW.C.OR4.r2 | 0.991695 | 321 | 451.263158 | 3.856507 |
| SW.C.OR4.r3 | 0.992692 | 314 | 390.56 | 3.883584 |
| SW.C.PW1.r1 | 0.994934 | 247 | 290.571429 | 3.665133 |
| SW.C.PW1.r2 | 0.993688 | 253 | 324.25 | 3.635511 |
| SW.C.PW1.r3 | 0.995017 | 264 | 305.162791 | 3.639925 |
| SW.C.PW2.r1 | 0.99427 | 267 | 325.65 | 3.476084 |
| SW.C.PW2.r2 | 0.99402 | 270 | 335.538462 | 3.461292 |
| SW.C.PW2.r3 | 0.993771 | 258 | 335.083333 | 3.66034 |
| SW.C.PW3.r1 | 0.997342 | 66 | 136.857143 | 1.564133 |
| SW.C.PW3.r2 | 0.997924 | 59 | 101.857143 | 1.60007 |
| SW.C.PW3.r3 | 0.997259 | 73 | 121 | 1.657334 |
| SW.C.PW4.r1 | 0.996429 | 234 | 257.153846 | 3.345135 |
| SW.C.PW4.r2 | 0.996014 | 225 | 260.25 | 3.368656 |
| SW.C.PW4.r3 | 0.9951 | 252 | 302.323529 | 3.608664 |
| SW.C.R.r1 | 0.993605 | 299 | 365.5 | 3.700091 |
| SW.C.R.r2 | 0.994768 | 273 | 332.181818 | 3.753607 |
| SW.C.R.r3 | 0.994851 | 278 | 330.527778 | 3.749432 |
| SW.N.E1.r1 | 0.988041 | 425 | 664.44186 | 4.30861 |
| SW.N.E1.r2 | 0.990366 | 384 | 520.122449 | 4.291112 |
| SW.N.E1.r3 | 0.989121 | 428 | 561.046875 | 4.29731 |
| SW.N.E2.r1 | 0.98937 | 367 | 526.372549 | 3.828168 |
| SW.N.E2.r2 | 0.990283 | 363 | 470.714286 | 3.915997 |
| SW.N.E2.r3 | 0.991197 | 358 | 465.019231 | 3.959259 |
| SW.N.ER1.r1 | 0.986961 | 429 | 590.131579 | 4.036562 |
| SW.N.ER1.r2 | 0.989785 | 427 | 528.391892 | 4.149008 |
| SW.N.ER1.r3 | 0.987875 | 453 | 621.015873 | 4.214559 |
| SW.N.ER2.r1 | 0.986961 | 416 | 613.516129 | 4.002655 |
| SW.N.ER2.r2 | 0.988041 | 400 | 574.508475 | 3.920815 |
| SW.N.ER2.r3 | 0.988207 | 423 | 581.904762 | 4.026354 |
| SW.N.ER3.r1 | 0.991031 | 364 | 471 | 3.92523 |
| SW.N.ER3.r2 | 0.993439 | 345 | 411.978261 | 3.905241 |
| SW.N.ER3.r3 | 0.993688 | 323 | 396.076923 | 3.833282 |
| SW.N.OPD1.r1 | 0.988124 | 448 | 599.537313 | 4.11865 |
| SW.N.OPD1.r2 | 0.989121 | 423 | 544.642857 | 4.079539 |
| SW.N.OPD1.r3 | 0.993439 | 407 | 452.308824 | 4.209104 |
| SW.N.OPD2.r1 | 0.989785 | 432 | 555 | 4.401488 |
| SW.N.OPD2.r2 | 0.989037 | 477 | 623.542373 | 4.627614 |
| SW.N.OPD2.r3 | 0.988705 | 448 | 581.043478 | 4.397949 |
| SW.N.OPD3.r1 | 0.989536 | 382 | 533.442308 | 3.627998 |
| SW.N.OPD3.r2 | 0.990698 | 385 | 496 | 3.63238 |
| SW.N.OPD3.r3 | 0.991031 | 377 | 486.018868 | 3.709681 |
| SW.N.OPD4.r1 | 0.990615 | 376 | 489 | 4.008003 |
| SW.N.OPD4.r2 | 0.989951 | 379 | 515.981132 | 4.095658 |
| SW.N.OPD4.r3 | 0.989619 | 390 | 523.62069 | 4.037977 |
| SW.N.OPD5.r1 | 0.985715 | 480 | 687.126761 | 4.350679 |
| SW.N.OPD5.r2 | 0.986214 | 468 | 669.397059 | 4.332427 |
| SW.N.OPD5.r3 | 0.988539 | 458 | 582.381579 | 4.304624 |
| SW.N.OR1.r1 | 0.991944 | 319 | 418.06383 | 3.788115 |
| SW.N.OR1.r2 | 0.991031 | 334 | 433.62069 | 3.748129 |
| SW.N.OR1.r3 | 0.991446 | 306 | 413.204082 | 3.710241 |
| SW.N.OR2.r1 | 0.992442 | 255 | 368.75 | 3.191841 |
| SW.N.OR2.r2 | 0.992442 | 276 | 378.375 | 3.346556 |
| SW.N.OR2.r3 | 0.99211 | 265 | 366.477273 | 3.237576 |
| SW.N.OR3.r1 | 0.99319 | 240 | 343.78125 | 3.421272 |
| SW.N.OR3.r2 | 0.995349 | 203 | 254.333333 | 3.224817 |
| SW.N.OR3.r3 | 0.992692 | 245 | 372.6 | 3.460134 |
| SW.N.OR4.r1 | 0.997343 | 261 | 299.886364 | 3.24342 |
| SW.N.OR4.r2 | 0.997342 | 201 | 212.022222 | 3.09807 |
| SW.N.OR4.r3 | 0.992609 | 253 | 342 | 3.209495 |
| SW.N.PW1.r1 | 0.994851 | 201 | 271.037037 | 2.470604 |
| SW.N.PW1.r2 | 0.99427 | 218 | 304.888889 | 2.664969 |
| SW.N.PW1.r3 | 0.995183 | 237 | 276.357143 | 2.993138 |
| SW.N.PW2.r1 | 0.992941 | 321 | 408.073171 | 3.828452 |
| SW.N.PW2.r2 | 0.992027 | 306 | 432.666667 | 3.729215 |
| SW.N.PW2.r3 | 0.992027 | 326 | 432.046512 | 3.936568 |
| SW.N.PW3.r1 | 0.991612 | 313 | 414 | 3.641101 |
| SW.N.PW3.r2 | 0.991114 | 301 | 432.883721 | 3.531688 |
| SW.N.PW3.r3 | 0.989702 | 314 | 509.538462 | 3.371676 |
| SW.N.PW4.r1 | 0.987792 | 431 | 574.08 | 4.120321 |
| SW.N.PW4.r2 | 0.988124 | 411 | 614.06 | 4.11249 |
| SW.N.PW4.r3 | 0.988622 | 406 | 569.438596 | 4.054915 |
| SW.N.R.r1 | 0.987875 | 509 | 682.52459 | 4.637006 |
| SW.N.R.r2 | 0.988788 | 510 | 633.90411 | 4.550524 |
| SW.N.R.r3 | 0.988788 | 513 | 644.086957 | 4.611595 |

**Table S3.** AMOVA statistics of beta diversity Morisita-Horn dissimilarity indices comparing pairwise microbiota of (**a**) average OR and ORF, and (**b**) independently replicate OR and ORF samples. *P*-value < 0.05 indicates statistical difference.

(**a**)

| **Pairwise area comparisons** | ***P*-value** |
| --- | --- |
| SW.C.OR vs. SW.N.OR | < 0.001 |
| SW.C.ORF vs. SW.C.OR | < 0.001 |
| SW.C.ORF vs. SW.N.OR | < 0.001 |

(**b**)

| **Pairwise sample comparisons** | ***P*-value** |
| --- | --- |
| SW.C.OR1 vs. SW.C.OR2 | 0.088 |
| SW.C.OR1 vs. SW.C.ORF1 | 0.124 |
| SW.C.OR1 vs. SW.C.ORF2 | 0.105 |
| SW.C.OR2 vs. SW.C.ORF1 | 0.095 |
| SW.C.OR2 vs. SW.C.ORF2 | 0.098 |
| SW.C.ORF1 vs. SW.C.ORF2 | 0.117 |

**Table S4.** Environmental and hospital characteristics of study sites.

| **Parameters** | **Hospital C** | **Hospital N** | **Sources** |
| --- | --- | --- | --- |
| **Geographic location** | | | |
| GPS coordinates | 13.7327°N, 100.5370°E | 16.7490°N, 100.1890°E | Google Maps |
| Province | Bangkok | Phitsanulok | — |
| Distance between hospitals | 339 km | | Google Earth Pro |
| **Urbanization indicators** | | | |
| Population density (people/km²) | 3,501 | 83.8 (province); 404.1 (urban district) | [37-40] |
| Land-use classification | Metropolitan/Urban (Commercial red zone) | Rural/Agricultural (Agricultural green zone) | [41,42] |
| Vegetation coverage (%) | 11.86 | 5.99 | R with OpenStreetMap |
| **Air quality (year 2022)** | | | |
| PM₁₀ annual mean ± SD (µg/m³) | 44.00 ± 9.49 | 41.83 ± 19.17 | [34] |
| PM₂.₅ annual mean ± SD (µg/m³) | 25.75 ± 6.59 | 22.67 ± 12.55 | [34] |
| **Hospital characteristics** | | | |
| Building | Bhumibol Adulyadej Building, King Chulalongkorn Memorial Hospital | Sirindhorn Building, Naresuan University Hospital | - |
| Hospital type | Super tertiary care | Super tertiary care | [43,44] |
| Registered outpatients (2022) | 13,281 | 103,325 | [43,44] |
| Annual outpatient visits (2022) | 1,841,234 | 362,480 | [43,44] |
| Annual inpatient admissions (2022) | 45,539 | 14,531 | [43,44] |
| Bed capacity | 1,427 | 485 | [43,44] |

**Table S5.** (**a**) Number of quality reads and (**b**) Good’s coverage and alpha diversity indices based on ASV pipeline results. Sample abbreviations (sample IDs) are: active air-pump samples or passive air-grille-swab samples (abbreviated A or SW) followed by hospital (King Chulalongkorn Memorial Hospital, C; or Naresuan University Hospital, N), areas (elevator, E; outpatient department, OPD; operating rooms, OR; HEPA-filter in operating rooms, ORF; patient wards, PW; restaurants, R; examination room, ER), and the replicate number.

(**a**)

| **Sample IDs** | **Quality reads** |
| --- | --- |
| A.C.r1 | 47,589 |
| A.C.r2 | 22,180 |
| A.N.r1 | 36,268 |
| A.N.r2 | 17,904 |
| **Sample IDs** | **Quality reads** |
| SW.C.OPD1.r1 | 53,419 |
| SW.C.OPD1.r2 | 51,985 |
| SW.C.OPD1.r3 | 24,522 |
| SW.C.R.r1 | 24,437 |
| SW.C.R.r2 | 25,013 |
| SW.C.R.r3 | 24,066 |
| SW.C.E1.r1 | 50,072 |
| SW.C.E1.r2 | 32,011 |
| SW.C.E2.r1 | 26,746 |
| SW.C.E2.r2 | 28,083 |
| SW.C.E2.r3 | 59,969 |
| SW.C.PW1.r1 | 28,709 |
| SW.C.PW1.r2 | 29,480 |
| SW.C.PW1.r3 | 23,355 |
| SW.C.PW2.r1 | 24,955 |
| SW.C.PW2.r2 | 20,896 |
| SW.C.PW2.r3 | 31,117 |
| SW.C.PW3.r1 | 21,609 |
| SW.C.PW3.r2 | 22,624 |
| SW.C.PW3.r3 | 24,607 |
| SW.C.PW4.r1 | 22,267 |
| SW.C.PW4.r2 | 26,596 |
| SW.C.PW4.r3 | 23,491 |
| SW.C.OR1.r1 | 24,093 |
| SW.C.OR1.r2 | 23,334 |
| SW.C.OR1.r3 | 28,240 |
| SW.C.OR2.r1 | 26,722 |
| SW.C.OR2.r2 | 24,914 |
| SW.C.OR2.r3 | 26,694 |
| SW.C.OR3.r1 | 40,903 |
| SW.C.OR3.r2 | 51,036 |
| SW.C.OR3.r3 | 40,683 |
| SW.C.OR4.r1 | 45,828 |
| SW.C.OR4.r2 | 59,673 |
| SW.C.OR4.r3 | 56,041 |
| SW.C.ORF1.r1 | 15,443 |
| SW.C.ORF1.r2 | 32,258 |
| SW.C.ORF1.r3 | 30,240 |
| SW.C.ORF2.r1 | 38,184 |
| SW.C.ORF2.r2 | 30,208 |
| SW.C.ORF2.r3 | 28,292 |
| SW.N.OPD1.r1 | 26,117 |
| SW.N.OPD1.r2 | 19,420 |
| SW.N.OPD1.r3 | 16,648 |
| SW.N.OPD2.r1 | 16,376 |
| SW.N.OPD2.r2 | 172,579 |
| SW.N.OPD2.r3 | 22,682 |
| SW.N.OPD3.r1 | 22,285 |
| SW.N.OPD3.r2 | 22,265 |
| SW.N.OPD3.r3 | 15,740 |
| SW.N.OPD4.r1 | 17,643 |
| SW.N.OPD4.r2 | 24,834 |
| SW.N.OPD4.r3 | 22,094 |
| SW.N.OPD5.r1 | 25,598 |
| SW.N.OPD5.r2 | 31,324 |
| SW.N.OPD5.r3 | 26,585 |
| SW.N.R.r1 | 16,479 |
| SW.N.R.r2 | 15,718 |
| SW.N.R.r3 | 16,968 |
| SW.N.E1.r1 | 26,488 |
| SW.N.E1.r2 | 12,025 |
| SW.N.E1.r3 | 27,784 |
| SW.N.E2.r1 | 20,434 |
| SW.N.E2.r2 | 20,948 |
| SW.N.E2.r3 | 27,823 |
| SW.N.PW1.r1 | 22,542 |
| SW.N.PW1.r2 | 55,002 |
| SW.N.PW1.r3 | 25,977 |
| SW.N.PW2.r1 | 23,611 |
| SW.N.PW2.r2 | 24,631 |
| SW.N.PW2.r3 | 22,895 |
| SW.N.PW3.r1 | 19,579 |
| SW.N.PW3.r2 | 16,956 |
| SW.N.PW3.r3 | 23,499 |
| SW.N.PW4.r1 | 21,715 |
| SW.N.PW4.r2 | 26,666 |
| SW.N.PW4.r3 | 47,851 |
| SW.N.OR1.r1 | 18,546 |
| SW.N.OR1.r2 | 47,622 |
| SW.N.OR1.r3 | 21,251 |
| SW.N.OR2.r1 | 18,776 |
| SW.N.OR2.r2 | 27,635 |
| SW.N.OR2.r3 | 20,284 |
| SW.N.OR3.r1 | 19,069 |
| SW.N.OR3.r2 | 9,812 |
| SW.N.OR3.r3 | 52,793 |
| SW.N.OR4.r1 | 10,146 |
| SW.N.OR4.r2 | 7,688 |
| SW.N.OR4.r3 | 28,977 |
| SW.N.ER1.r1 | 100,077 |
| SW.N.ER1.r2 | 26,200 |
| SW.N.ER1.r3 | 23,860 |
| SW.N.ER2.r1 | 24,160 |
| SW.N.ER2.r2 | 19,561 |
| SW.N.ER2.r3 | 25,390 |
| SW.N.ER3.r1 | 26,190 |
| SW.N.ER3.r2 | 19,406 |
| SW.N.ER3.r3 | 22,202 |

(**b**)

| **Sample IDs** | **Good's coverage** | **No. of OTUs** | **Chao1** | **Shannon** |
| --- | --- | --- | --- | --- |
| A.C.r1 | 0.179381 | 8451 | 1246200 | 8.538212 |
| A.C.r2 | 0.109994 | 9107 | 4538655 | 8.85547 |
| A.N.r1 | 0.122511 | 8986 | 2485689 | 8.745193 |
| A.N.r2 | 0.062586 | 9535 | 15084470 | 8.955678 |
| SW.C.ORF1.r1 | 0.004435 | 10108 | 25515133 | 9.21511 |
| SW.C.ORF1.r2 | 0.054997 | 9657 | 2007921 | 9.083043 |
| SW.C.ORF1.r3 | 0.061009 | 9605 | 2072201 | 9.071001 |
| SW.C.ORF2.r1 | 0.138183 | 8828 | 2257322 | 8.675059 |
| SW.C.ORF2.r2 | 0.130593 | 8894 | 3545313 | 8.708681 |
| SW.C.ORF2.r3 | 0.122117 | 8968 | 3314207 | 8.731861 |
| SW.C.E1.r1 | 0.095801 | 9281 | 2484372 | 8.97501 |
| SW.C.E1.r2 | 0.030455 | 9876 | 9685549 | 9.153502 |
| SW.C.E2.r1 | 0.033806 | 9842 | 9618743 | 9.142393 |
| SW.C.E2.r2 | 0.035285 | 9827 | 23958616 | 9.136666 |
| SW.C.E2.r3 | 0.101124 | 9245 | 2319392 | 8.956415 |
| SW.C.OPD1.r1 | 0.068993 | 9565 | 1248697 | 9.080719 |
| SW.C.OPD1.r2 | 0.065149 | 9608 | 1225226 | 9.097016 |
| SW.C.OPD1.r3 | 0.014193 | 10025 | 12513775 | 9.196769 |
| SW.C.OR1.r1 | 0.030357 | 9876 | 16139277 | 9.152979 |
| SW.C.OR1.r2 | 0.029273 | 9882 | 16175374 | 9.152445 |
| SW.C.OR1.r3 | 0.032427 | 9858 | 8040164 | 9.147644 |
| SW.C.OR2.r1 | 0.026513 | 9912 | 12203069 | 9.167026 |
| SW.C.OR2.r2 | 0.015277 | 10014 | 16645029 | 9.193072 |
| SW.C.OR2.r3 | 0.025034 | 9927 | 8163408 | 9.17043 |
|  |  |  |  |  |
| SW.C.OR3.r1 | 0.074118 | 9506 | 1927720 | 9.061276 |
| SW.C.OR3.r2 | 0.082594 | 9433 | 1978287 | 9.045586 |
| SW.C.OR3.r3 | 0.077568 | 9466 | 2928850 | 9.047268 |
| SW.C.OR4.r1 | 0.066036 | 9575 | 1672262 | 9.072918 |
| SW.C.OR4.r2 | 0.090085 | 9367 | 1587526 | 9.019666 |
| SW.C.OR4.r3 | 0.092844 | 9345 | 1254998 | 9.011449 |
| SW.C.PW1.r1 | 0.031737 | 9873 | 4396289 | 9.155806 |
| SW.C.PW1.r2 | 0.042283 | 9774 | 6753372 | 9.124677 |
| SW.C.PW1.r3 | 0.035778 | 9817 | 47858470 | 9.13254 |
| SW.C.PW2.r1 | 0.031342 | 9857 | 16106483 | 9.13989 |
| SW.C.PW2.r2 | 0.047309 | 9717 | 6682709 | 9.103228 |
| SW.C.PW2.r3 | 0.04386 | 9759 | 5237520 | 9.124169 |
| SW.C.PW3.r1 | 0.061404 | 9542 | 15122543 | 8.966219 |
| SW.C.PW3.r2 | 0.061305 | 9544 | 15125719 | 8.973173 |
| SW.C.PW3.r3 | 0.068401 | 9476 | 22342189 | 8.946673 |
| SW.C.PW4.r1 | 0.055391 | 9620 | 6569868 | 9.019212 |
| SW.C.PW4.r2 | 0.05411 | 9631 | 23032834 | 9.02903 |
| SW.C.PW4.r3 | 0.032624 | 9840 | 9642281 | 9.127554 |
| SW.C.R.r1 | 0.037256 | 9806 | 9550212 | 9.119299 |
| SW.C.R.r2 | 0.012616 | 10031 | 25097608 | 9.192643 |
| SW.C.R.r3 | 0.030455 | 9867 | 16135989 | 9.142478 |
| SW.N.E1.r1 | 0.007688 | 10081 | 12679401 | 9.209878 |
| SW.N.E1.r2 | 0.009955 | 10059 | 25233054 | 9.203485 |
| SW.N.E1.r3 | 0.009856 | 10063 | 12624072 | 9.205308 |
| SW.N.E2.r1 | 0.019318 | 9971 | 9909226 | 9.173228 |
| SW.N.E2.r2 | 0.020402 | 9960 | 24703406 | 9.171792 |
| SW.N.E2.r3 | 0.078652 | 9426 | 4378214 | 9.00506 |
| SW.N.ER1.r1 | 0.06919 | 9559 | 1124305 | 9.068403 |
| SW.N.ER1.r2 | 0.019022 | 9978 | 7085139 | 9.181608 |
| SW.N.ER1.r3 | 0.023655 | 9934 | 9821827 | 9.168043 |
| SW.N.ER2.r1 | 0.017445 | 9990 | 9947089 | 9.18206 |
| SW.N.ER2.r2 | 0.018037 | 9981 | 24822833 | 9.178617 |
| SW.N.ER2.r3 | 0.027696 | 9892 | 24336982 | 9.155401 |
| SW.N.ER3.r1 | 0.013799 | 10023 | 16695028 | 9.192576 |
| SW.N.ER3.r2 | 0.010349 | 10049 | 50415869 | 9.196991 |
| SW.N.ER3.r3 | 0.016164 | 10001 | 12463794 | 9.184968 |
| SW.N.OPD1.r1 | 0.013207 | 10028 | 25067561 | 9.194553 |
| SW.N.OPD1.r2 | 0.012419 | 10034 | 25107629 | 9.193783 |
| SW.N.OPD1.r3 | 0.012813 | 10029 | 50165149 | 9.193254 |
| SW.N.OPD2.r1 | 0.002661 | 10124 | 25606135 | 9.220083 |
| SW.N.OPD2.r2 | 0.075202 | 9553 | 546329.3 | 9.087935 |
| SW.N.OPD2.r3 | 0.023458 | 9943 | 7021268 | 9.174302 |
| SW.N.OPD3.r1 | 0.014686 | 10010 | 24992513 | 9.18201 |
| SW.N.OPD3.r2 | 0.011236 | 10043 | 25167791 | 9.195611 |
| SW.N.OPD3.r3 | 0.005519 | 10097 | 50909102 | 9.212799 |
| SW.N.OPD4.r1 | 0.014587 | 10021 | 16668355 | 9.19603 |
| SW.N.OPD4.r2 | 0.009758 | 10063 | 12626583 | 9.205787 |
| SW.N.OPD4.r3 | 0.020304 | 9960 | 24708375 | 9.171182 |
| SW.N.OPD5.r1 | 0.013109 | 10031 | 50135109 | 9.196475 |
| SW.N.OPD5.r2 | 0.016164 | 10004 | 24917590 | 9.190107 |
| SW.N.OPD5.r3 | 0.018529 | 9982 | 16535283 | 9.18254 |
| SW.N.OR1.r1 | 0.014587 | 10021 | 8339188 | 9.195128 |
| SW.N.OR1.r2 | 0.045141 | 9750 | 3920069 | 9.123025 |
| SW.N.OR1.r3 | 0.028878 | 9885 | 16188511 | 9.154578 |
| SW.N.OR2.r1 | 0.04386 | 9737 | 11772200 | 9.101306 |
| SW.N.OR2.r2 | 0.036566 | 9813 | 9563898 | 9.129388 |
| SW.N.OR2.r3 | 0.020796 | 9965 | 16459013 | 9.177848 |
| SW.N.OR3.r1 | 0.034496 | 9829 | 23997784 | 9.131167 |
| SW.N.OR3.r2 | 0.033314 | 9861 | 4382000 | 9.153436 |
| SW.N.OR3.r3 | 0.026513 | 9893 | 48782519 | 9.14533 |
| SW.N.OR4.r1 | 0.036468 | 9,815 | 9565855 | 9.131119 |
| SW.N.OR4.r2 | 0.062685 | 9,535 | 11313359 | 8.980849 |
| SW.N.OR4.r3 | 0.131480 | 8,903 | 2435232 | 8.728779 |
| SW.N.PW1.r1 | 0.055391 | 9,615 | 6569863 | 9.021541 |
| SW.N.PW1.r2 | 0.020402 | 9,968 | 16472265 | 9.181701 |
| SW.N.PW1.r3 | 0.018628 | 9,978 | 24792951 | 9.177552 |
| SW.N.PW2.r1 | 0.020895 | 9,958 | 16455695 | 9.176581 |
| SW.N.PW2.r2 | 0.019318 | 9,967 | 24758105 | 9.168934 |
| SW.N.PW2.r3 | 0.024246 | 9,916 | 24509941 | 9.141582 |
| SW.N.PW3.r1 | 0.027006 | 9,895 | 12190709 | 9.139318 |
| SW.N.PW3.r2 | 0.022669 | 9,947 | 12299590 | 9.175213 |
| SW.N.PW3.r3 | 0.022570 | 9,950 | 5473115 | 9.175851 |
| SW.N.PW4.r1 | 0.066824 | 9,559 | 2997344 | 9.068351 |
| SW.N.PW4.r2 | 0.004337 | 10,111 | 25520187 | 9.217407 |
| SW.N.PW4.r3 | 0.003154 | 10,118 | 51151559 | 9.218121 |
| SW.N.R.r1 | 0.001380 | 10,136 | 25671959 | 9.222989 |
| SW.N.R.r2 | 0.179381 | 8451 | 1246200 | 8.538212 |
| SW.N.R.r3 | 0.109994 | 9107 | 4538655 | 8.85547 |

**Table S6.** Weighted NSTI values, representing the average phylogenetic distance between the OTUs in a sample and the closest reference genome. NSTI < 0.15 (for environmental datasets) indicates a higher degree of accuracy in the functional metabolic profiles predicted by PICRUSt.

| **Groups** | **Weighted NSTI** | **Groups** | **Weighted NSTI** |
| --- | --- | --- | --- |
| **Hospital C** |  | **Hospital N** |  |
| A.C.r1 | 0.0900 | A.N.r1 | 0.0526 |
| A.C.r2 | 0.0841 | A.N.r2 | 0.0281 |
| SW.C.ORF1.r1 | 0.1244 | SW.N.E1.r1 | 0.1271 |
| SW.C.ORF1.r2 | 0.1265 | SW.N.E1.r2 | 0.1273 |
| SW.C.ORF1.r3 | 0.1255 | SW.N.E1.r3 | 0.1276 |
| SW.C.ORF2.r1 | 0.1268 | SW.N.E2.r1 | 0.1306 |
| SW.C.ORF2.r2 | 0.1279 | SW.N.E2.r2 | 0.1306 |
| SW.C.ORF2.r3 | 0.1273 | SW.N.E2.r3 | 0.1309 |
| SW.C.E1.r1 | 0.1305 | SW.N.ER1.r1 | 0.1291 |
| SW.C.E1.r2 | 0.1309 | SW.N.ER1.r2 | 0.1293 |
| SW.C.E2.r1 | 0.1309 | SW.N.ER1.r3 | 0.1293 |
| SW.C.E2.r2 | 0.1309 | SW.N.ER2.r1 | 0.1295 |
| SW.C.E2.r3 | 0.1314 | SW.N.ER2.r2 | 0.1296 |
| SW.C.OPD1.r1 | 0.1279 | SW.N.ER2.r3 | 0.1299 |
| SW.C.OPD1.r2 | 0.1278 | SW.N.ER3.r1 | 0.1304 |
| SW.C.OPD1.r3 | 0.1276 | SW.N.ER3.r2 | 0.1308 |
| SW.C.OR1.r1 | 0.1311 | SW.N.ER3.r3 | 0.1294 |
| SW.C.OR1.r2 | 0.1311 | SW.N.OPD1.r1 | 0.1296 |
| SW.C.OR1.r3 | 0.1312 | SW.N.OPD1.r2 | 0.1296 |
| SW.C.OR2.r1 | 0.1305 | SW.N.OPD1.r3 | 0.1300 |
| SW.C.OR2.r2 | 0.1296 | SW.N.OPD2.r1 | 0.1288 |
| SW.C.OR2.r3 | 0.1299 | SW.N.OPD2.r2 | 0.1284 |
| SW.C.OR3.r1 | 0.1291 | SW.N.OPD2.r3 | 0.1297 |
| SW.C.OR3.r2 | 0.1288 | SW.N.OPD3.r1 | 0.1259 |
| SW.C.OR3.r3 | 0.1290 | SW.N.OPD3.r2 | 0.1243 |
| SW.C.OR4.r1 | 0.1288 | SW.N.OPD3.r3 | 0.1285 |
| SW.C.OR4.r2 | 0.1289 | SW.N.OPD4.r1 | 0.1297 |
| SW.C.OR4.r3 | 0.1291 | SW.N.OPD4.r2 | 0.1298 |
| SW.C.PW1.r1 | 0.1288 | SW.N.OPD4.r3 | 0.1302 |
| SW.C.PW1.r2 | 0.1290 | SW.N.OPD5.r1 | 0.1290 |
| SW.C.PW1.r3 | 0.1311 | SW.N.OPD5.r2 | 0.1294 |
| SW.C.PW2.r1 | 0.1315 | SW.N.OPD5.r3 | 0.1293 |
| SW.C.PW2.r2 | 0.1316 | SW.N.OR1.r1 | 0.1291 |
| SW.C.PW2.r3 | 0.1294 | SW.N.OR1.r2 | 0.1299 |
| SW.C.PW3.r1 | 0.0228 | SW.N.OR1.r3 | 0.1300 |
| SW.C.PW3.r2 | 0.0190 | SW.N.OR2.r1 | 0.1309 |
| SW.C.PW3.r3 | 0.0193 | SW.N.OR2.r2 | 0.1307 |
| SW.C.PW4.r1 | 0.1254 | SW.N.OR2.r3 | 0.1307 |
| SW.C.PW4.r2 | 0.1241 | SW.N.OR3.r1 | 0.1299 |
| SW.C.PW4.r3 | 0.1301 | SW.N.OR3.r2 | 0.1304 |
| SW.C.R.r1 | 0.1263 | SW.N.OR3.r3 | 0.1300 |
| SW.C.R.r2 | 0.1268 | SW.N.OR4.r1 | 0.1314 |
| SW.C.R.r3 | 0.1270 | SW.N.OR4.r2 | 0.1317 |
|  |  | SW.N.OR4.r3 | 0.1312 |
|  |  | SW.N.PW1.r1 | 0.1169 |
|  |  | SW.N.PW1.r2 | 0.1196 |
|  |  | SW.N.PW1.r3 | 0.1240 |
|  |  | SW.N.PW2.r1 | 0.1306 |
|  |  | SW.N.PW2.r2 | 0.1312 |
|  |  | SW.N.PW2.r3 | 0.1302 |
|  |  | SW.N.PW3.r1 | 0.1271 |
|  |  | SW.N.PW3.r2 | 0.1272 |
|  |  | SW.N.PW3.r3 | 0.1275 |
|  |  | SW.N.PW4.r1 | 0.1295 |
|  |  | SW.N.PW4.r2 | 0.1298 |
|  |  | SW.N.PW4.r3 | 0.1299 |
|  |  | SW.N.R.r1 | 0.1240 |
|  |  | SW.N.R.r2 | 0.1254 |
|  |  | SW.N.R.r3 | 0.1257 |
| **Mean ± SD** | **0.1193 ± 0.0289** | **Mean ± SD** | **0.1257± 0.0165** |

**Table S7.** Comparative land use density. 'n' represents the total number of individual land-use polygons identified within a 5-km radius of each hospital using the OpenStreetMap database. The percentages in parentheses indicate the relative proportion of each land-use category relative to the total number of polygons at that site.

| **Land Use Category** | **Sub-type** | **Hospital C [n = 3,275]** | **Hospital N [n = 122]** |
| --- | --- | --- | --- |
| 1. Urban/Residential | residential | 1,069 (32.6%) | 10 (8.2%) |
| 2. Commercial & Retail | commercial, retail | 799 (24.4%) | 0 (0.0%) |
| 3. Agricultural Land | farmland, orchard | 75 (2.3%) | 65 (53.3%) |
| 4. Natural Vegetation | forest, grass, meadow | 985 (30.1%) | 22 (18.0%) |
| 5. Industrial/Construction | construction, industrial, brownfield | 292 (8.9%) | 2 (1.6%) |
| 6. Others (Special Use) | religious, cemetery, reservoir, quarry | 55 (1.7%) | 23 (18.9%) |
| **Total** |  | **100%** | **100%** |

**Table S8.** PERMDISP testing for homogeneity dispersions of Morisita–Horn dissimilarity matrices beta diversity (*P*‑values were calculated by ANOVA with 999 permutations, and *P* > 0.05 indicates homogeneous dispersion).

| **Taxonomic level** | **Comparison group** | **Figure reference** | **F-value** | ***P*-value (Pr>F)** |
| --- | --- | --- | --- | --- |
| **Genus level** | Overall Hospital | Fig. 3a | 0.0005 | 0.9822 |
|  | Overall Department | Fig. 3b | 0.6517 | 0.6656 |
|  | Department: OR | Fig. 3c | 2.1072 | 0.1577 |
|  | Department: OPD | Fig. 3c | 1.0974 | 0.3104 |
|  | Department: PW | Fig. 3c | 0.0027 | 0.9589 |
|  | Department: E | Fig. 3c | 1.4265 | 0.2629 |
|  | Department: R | Fig. 3c | 0.0001 | 0.9913 |
|  | Department: Air | Fig. 3c | 2.47 × 10^30^ | < 2.2 × 10^-16^ |
| **Species Level** | Overall Hospital | - | 0.7408 | 0.3914 |
|  | Overall Department | Fig. 4a,4b | 1.0681 | 0.3830 |
|  | Department: OR | Fig. 6b | 0.0427 | 0.9583 |
