# Supplementary figures and images for: Air Bacterial Microbiomes in Hospitals: Case Studies from a Metropolis and a Small City of Thailand
Source: Comput Struct Biotechnol J. 2026 Apr 29;35(1):0068. doi: 10.34133/csbj.0068 (PMC13125743; doi:10.34133/csbj.0068)

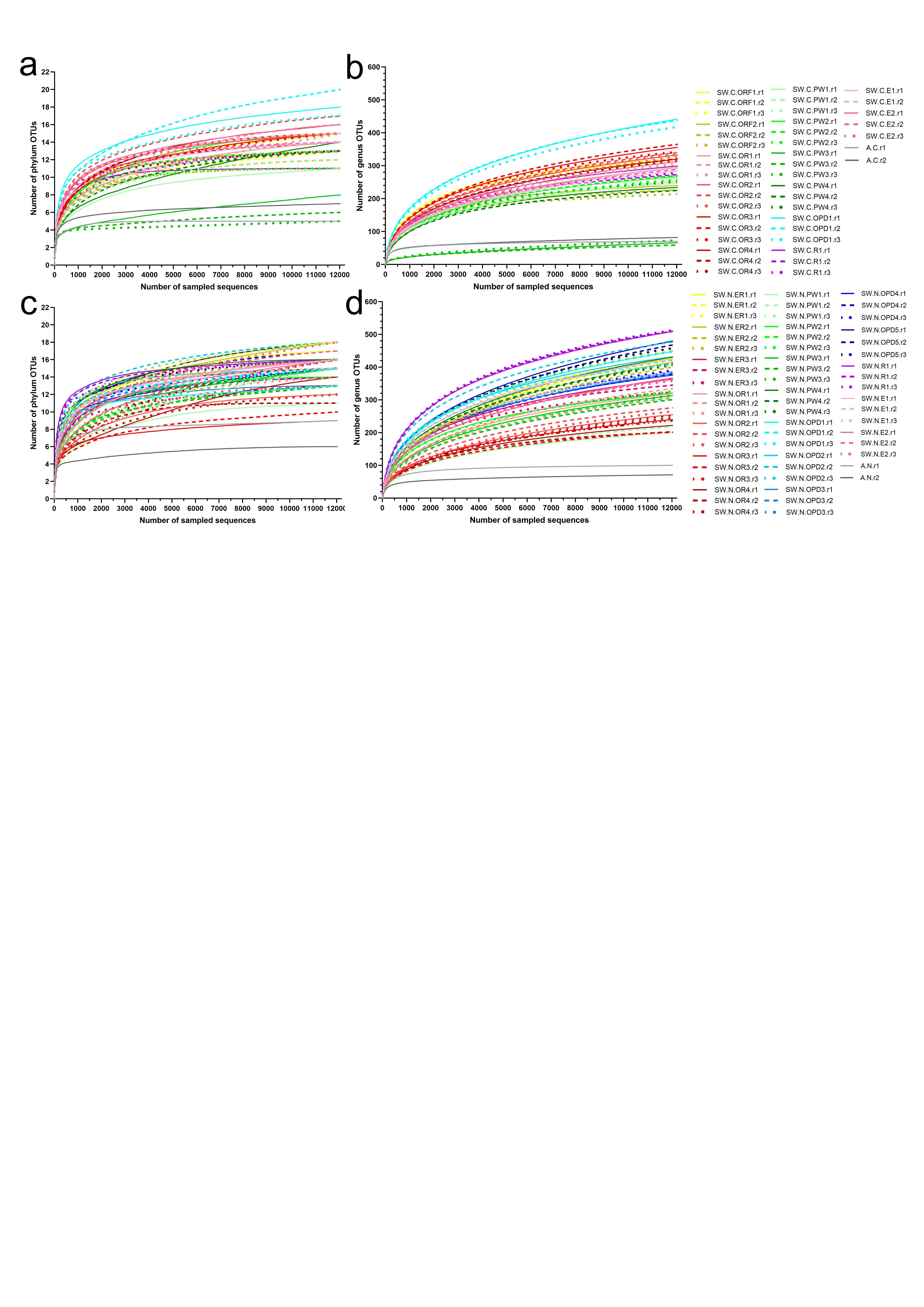

Supplement: Supplementary 1 — Figs. S1 to S7 Tables S1 to S8 [file csbj.0068.f1.zip › Fig. S1.jpg]

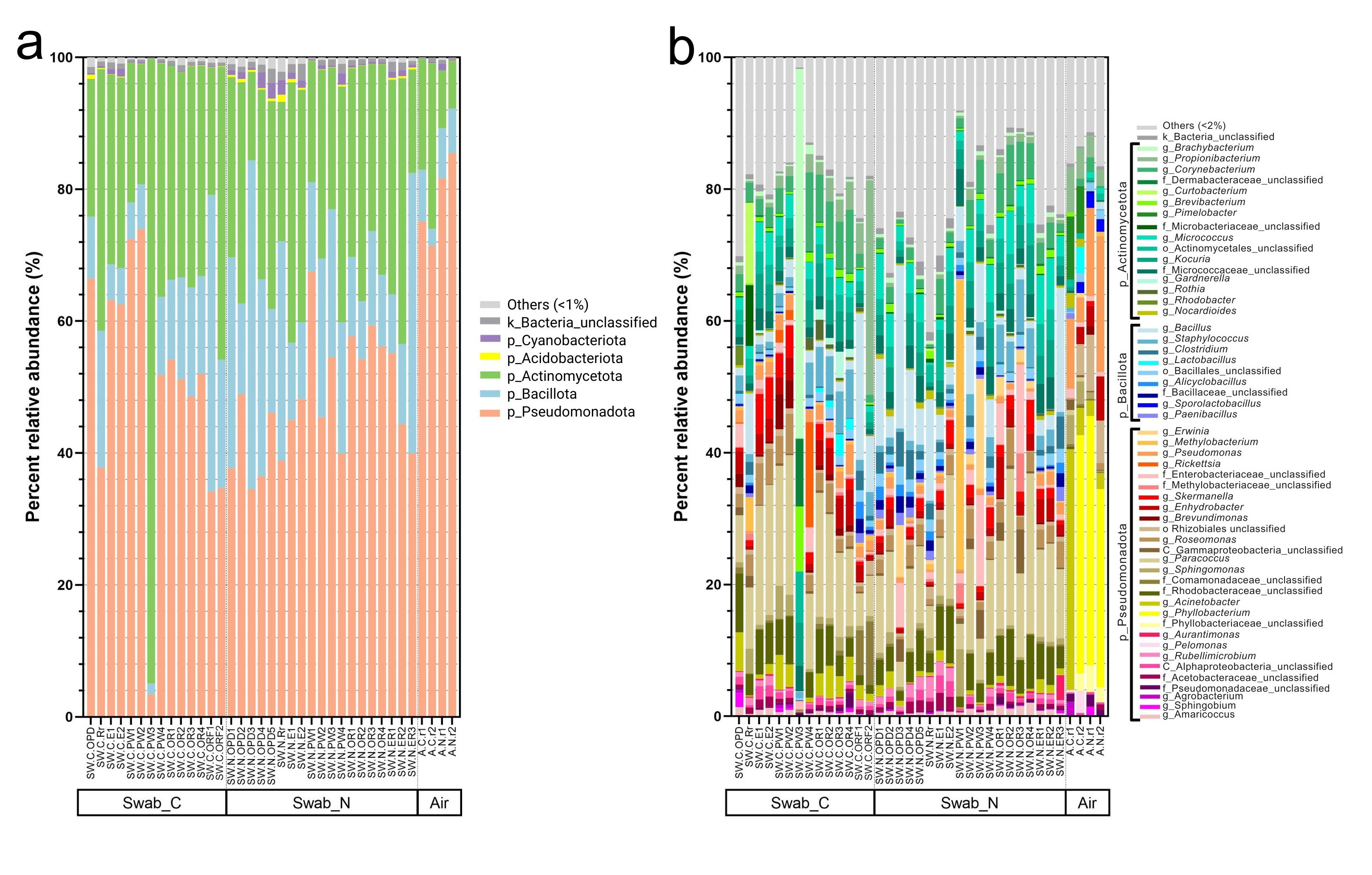

Supplement: Supplementary 1 — Figs. S1 to S7 Tables S1 to S8 [file csbj.0068.f1.zip › FigS2.jpg]

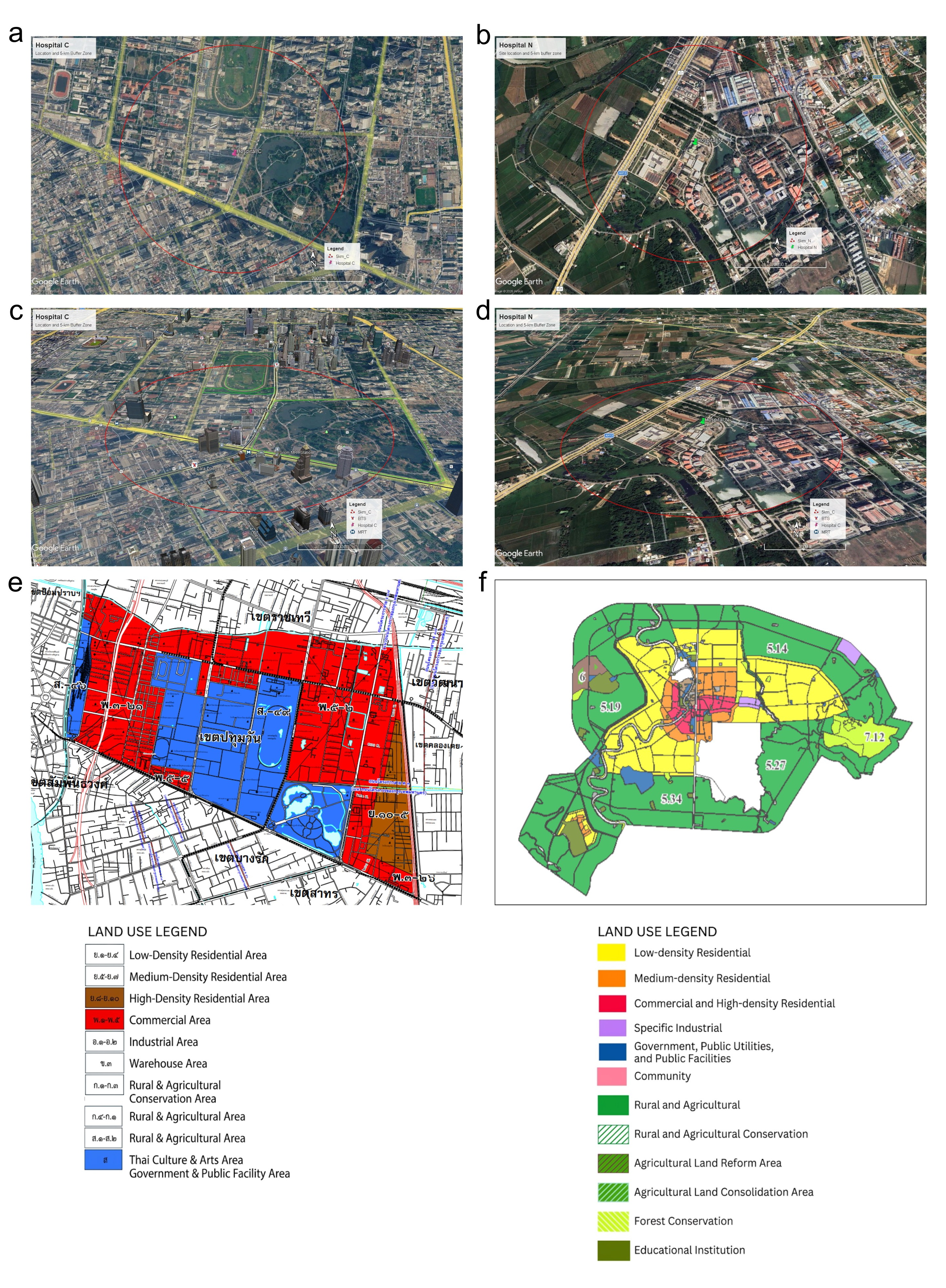

Supplement: Supplementary 1 — Figs. S1 to S7 Tables S1 to S8 [file csbj.0068.f1.zip › FigS3.jpg]

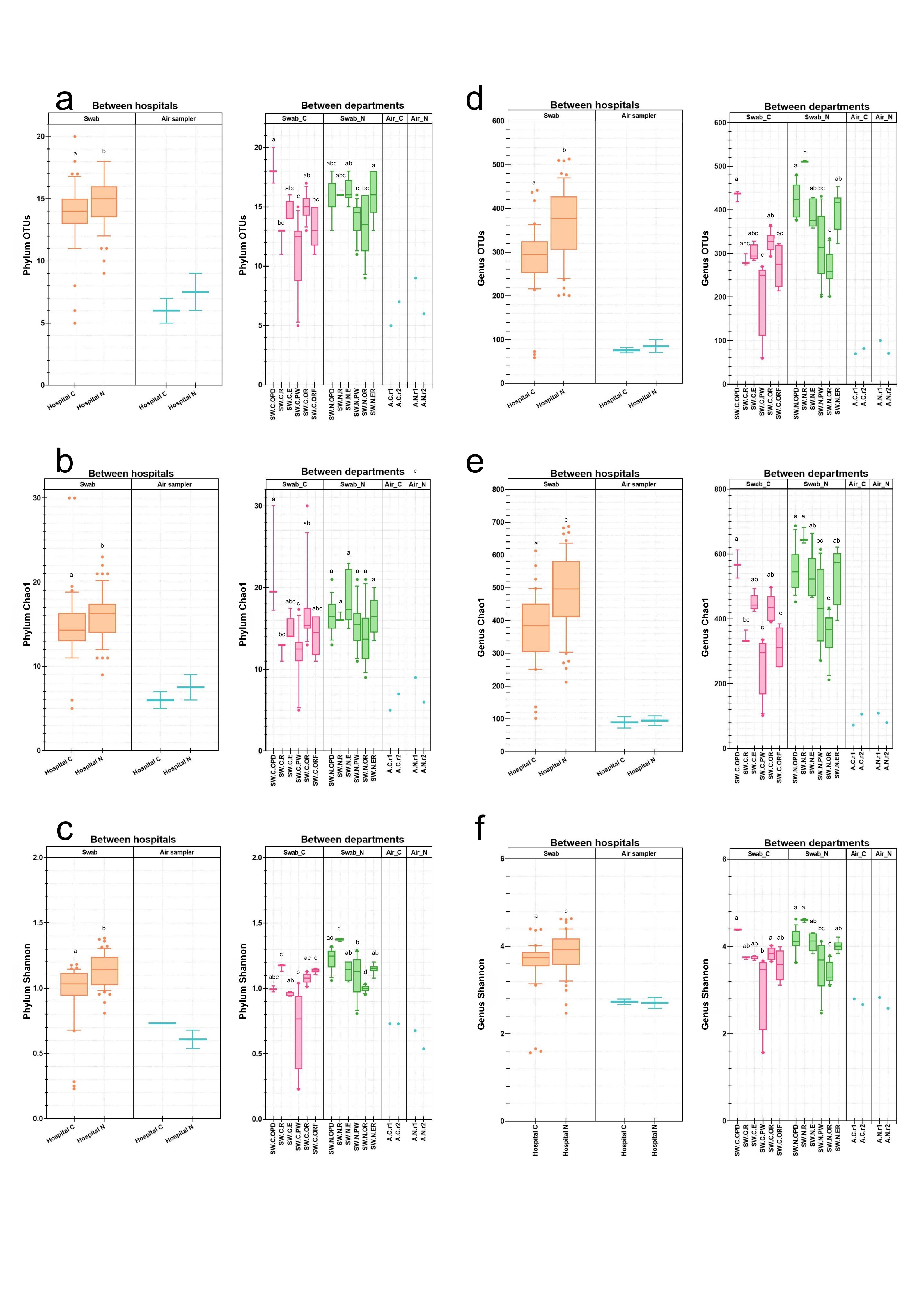

Supplement: Supplementary 1 — Figs. S1 to S7 Tables S1 to S8 [file csbj.0068.f1.zip › FigS4.jpg]

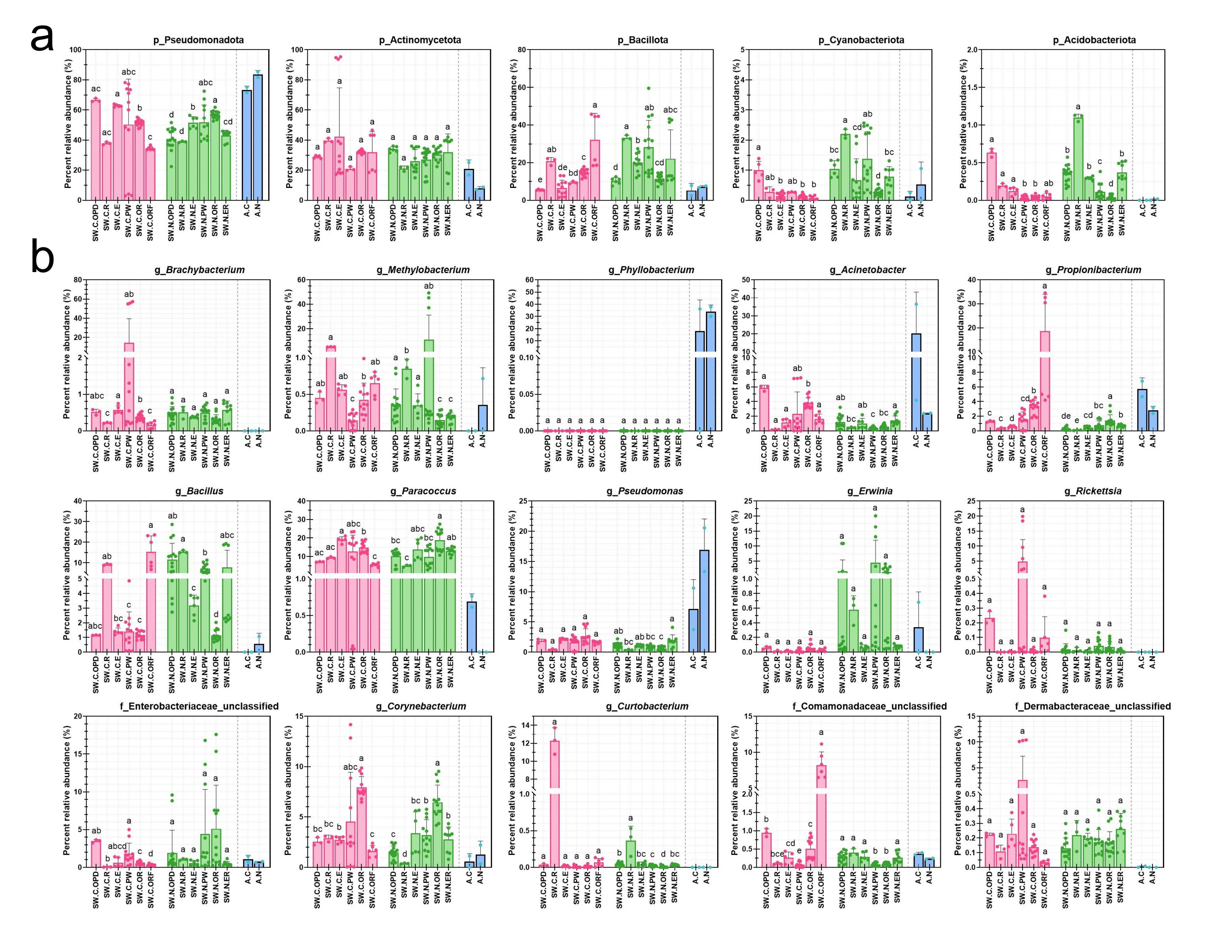

Supplement: Supplementary 1 — Figs. S1 to S7 Tables S1 to S8 [file csbj.0068.f1.zip › FigS5.jpg]

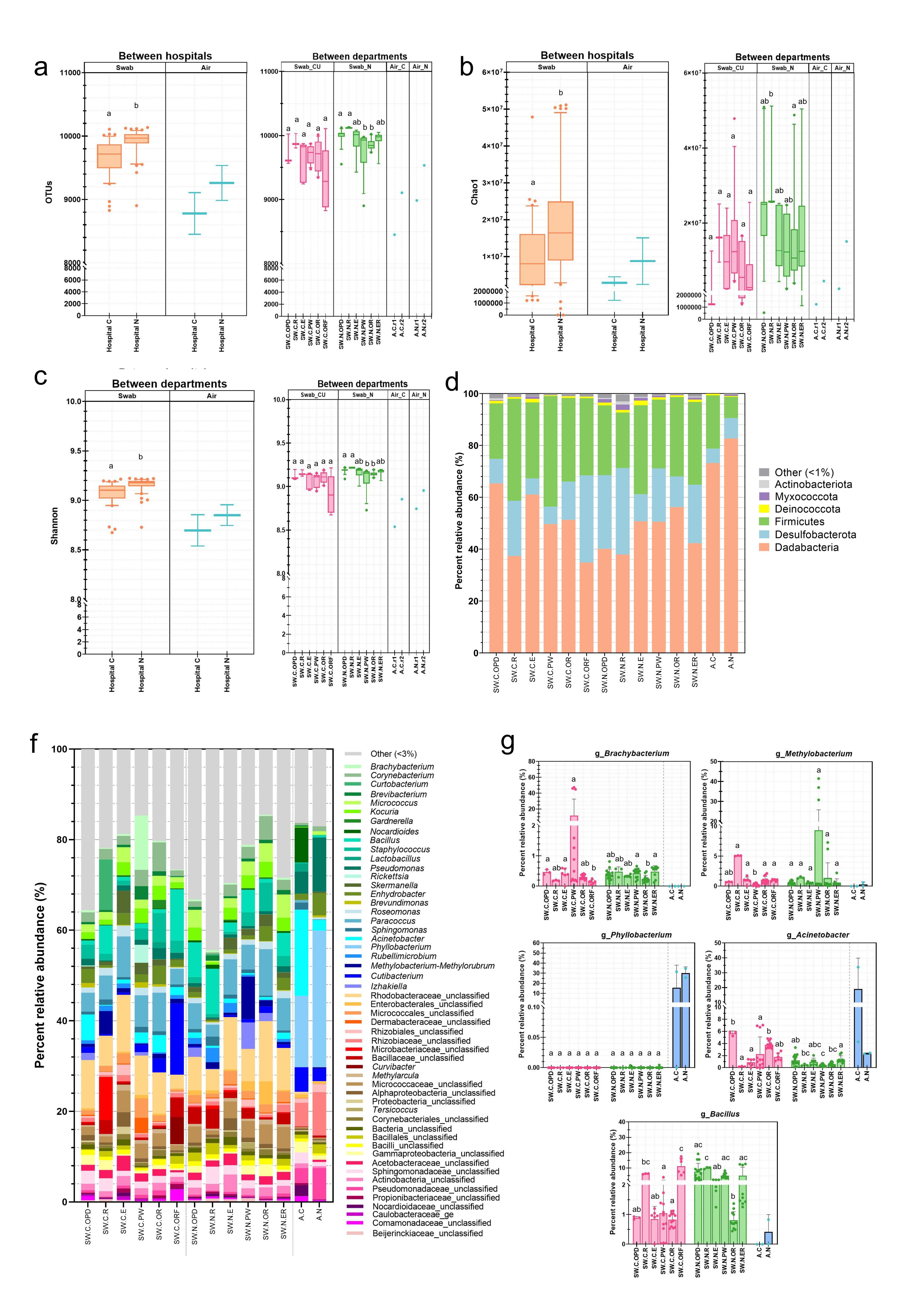

Supplement: Supplementary 1 — Figs. S1 to S7 Tables S1 to S8 [file csbj.0068.f1.zip › FigS6.jpg]

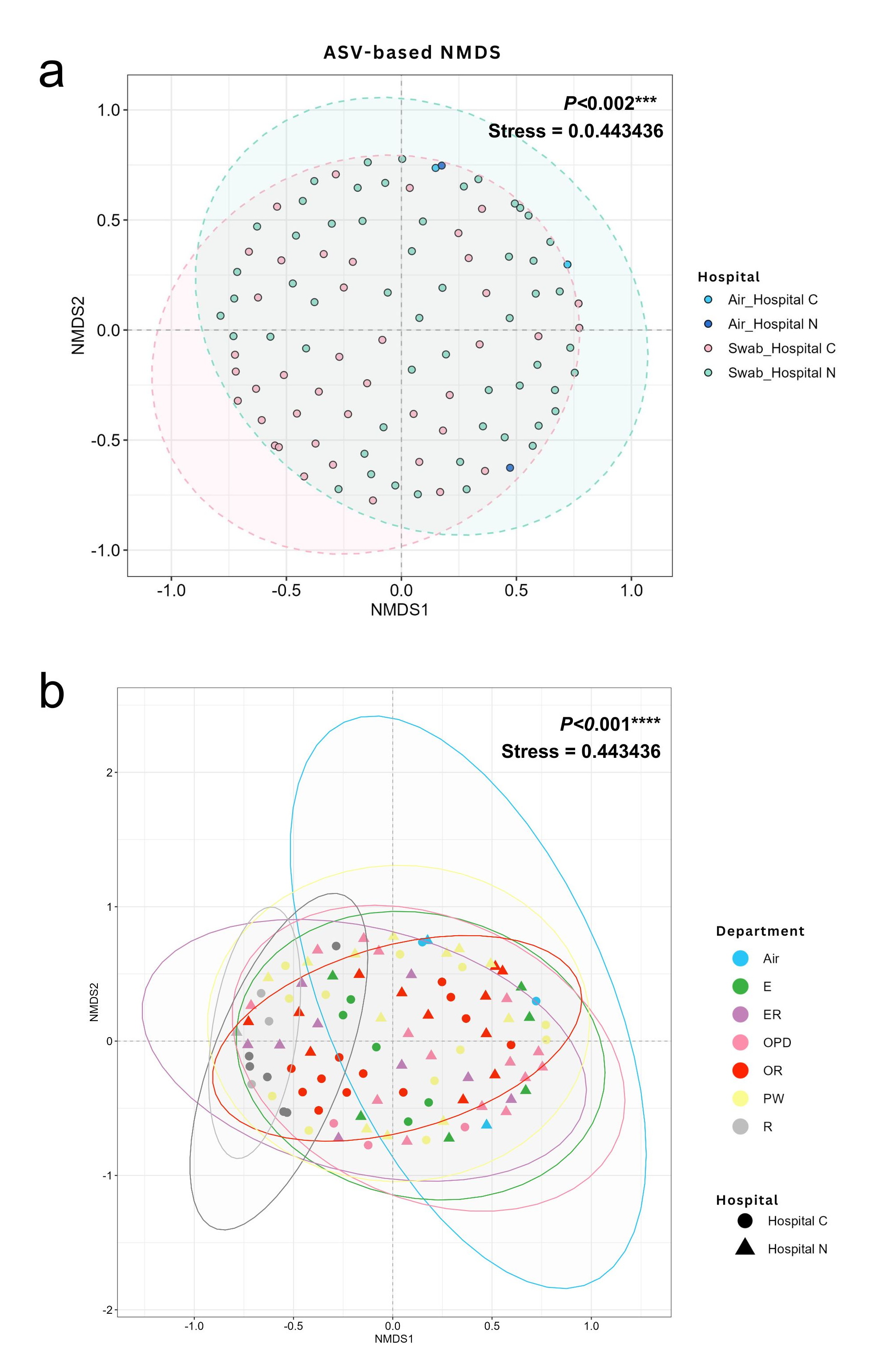

Supplement: Supplementary 1 — Figs. S1 to S7 Tables S1 to S8 [file csbj.0068.f1.zip › FigS7.jpg]
